# Interrogating a Multifactorial Model of Breast Conserving Therapy with Clinical Data

**DOI:** 10.1371/journal.pone.0125006

**Published:** 2015-04-23

**Authors:** Remi Salmon, Marc Garbey, Linda W. Moore, Barbara L. Bass

**Affiliations:** 1 Department of Computer Science, University of Houston, Houston, TX, USA; 2 Department of Biology and Biochemistry, University of Houston, Houston, TX, USA; 3 Methodist Institute for Technology, Innovation and Education, Houston, TX, USA; 4 Department of Surgery, Houston Methodist, Houston, TX, USA; University Campus Bio-Medico, ITALY

## Abstract

Most women with early stage breast cancer do not require removal of the entire breast to treat their cancer; instead, up to 70% of women can be effectively and safely treated by breast conserving therapy (BCT) with surgical removal of the tumor only (lumpectomy) followed by radiation treatment of the remaining breast tissue. Unfortunately, the final contour and cosmesis of the treated breast is suboptimal in approximately 30% of patients. The ability to accurately predict breast contour after BCT for breast cancer could significantly improve patient decision-making regarding the choice of surgery for breast cancer. Our overall hypothesis is that the complex interplay among mechanical forces due to gravity, breast tissue constitutive law distribution, inflammation induced by radiotherapy and internal stress generated by the healing process play a dominant role in determining the success or failure of lumpectomy in preserving the breast contour and cosmesis. We have shown here from a first patient study that even in the idealistic situation of excellent cosmetic outcome this problem requires multiscale modeling. We propose a method to decide which component of the model works best for each phase of healing and what parameters should be considered dominant and patient specific. This patient study is part of a clinical trial registered on ClinicalTrial.gov, identifier NCT02310711.

## Introduction

Breast cancer is the most common cancer of women in the world. It is estimated that one woman out of eight will be exposed to breast cancer during her lifetime [1]. Improving breast cancer treatment outcome and survival depends on early detection and effective use of multimodality therapy: surgery, radiation oncology, hormonal and chemotherapy treatments [2]. Most women with early stage breast cancer do not require removal of the entire breast (mastectomy) to treat their cancer; instead, up to 70% of women can be effectively and safely treated by breast conserving therapy (BCT) with surgical removal of the tumor only (lumpectomy) followed by radiation treatment of the remaining breast tissue [3, 4]. The goals of BCT are to achieve local control of the cancer as well as to preserve a breast that satisfies the woman’s cosmetic, emotional and physical needs. Unfortunately, the final contour and cosmesis of the treated breast is suboptimal in approximately 30% of patients [5, 6].

Our goal is to determine if patterns of deformity can be predicted by multiscale mathematical models based on preoperative imaging and surgical data points [7]. Research efforts to improve the surgical outcomes of breast conserving therapy in regards to prediction of cosmetic and functional outcome are very limited. To our knowledge we are the first team working on a computational framework designed to predict BCT outcomes and explore targets for improvement. The ability to accurately predict breast contour after breast conserving therapy for breast cancer could significantly improve patient decision-making regarding the choice of surgery for breast cancer—BCT or mastectomy [8, 9]. As BCT is equivalent to total mastectomy to achieve overall survival in patients with non-metastatic breast cancer, the procedure of BCT offers significant advantages for the 70% of patients who are candidates for this procedure.

There are currently no tools, other than surgical experience and judgment that can optimize cosmetic outcome or predict the impact of partial mastectomy on the contour and deformity of the treated breast. This focus of our research goes beyond classical tissue mechanics and incorporates novel important variables into the model including tissue plasticity and the dynamics of tissue healing and repair, both primarily and in the setting of radiation therapy. Our overall hypothesis is that the complex interplay among mechanical forces due to gravity, breast tissue constitutive law distribution, inflammation induced by radiotherapy and internal stress generated by the healing process play a dominant role in determining the success or failure of lumpectomy in preserving the breast shape and cosmesis.

We have built a general computational framework to test this hypothesis [10–13]. Our model encompasses multiple scales in space (from cells to tissue) and time (from minutes for the tissue mechanics to months for healing). We have used a modular method coupling mathematical models and corresponding software for patient specific data to test our hypothesis and refine the model.

From that conceptual work many scenarios seem possible. However we do not know *a priori* which *factors are dominant*, nor the *optimal clinical protocol* for validating the model. In other words, we do not know precisely what medical imaging is needed and when should we acquire the data in order to control cost, spare our patients from unnecessary medical exam and still get enough information to calibrate the model. Additionally, a typical pilot study to follow a patient requires at least six months and, more preferably, a year. To our knowledge, no animal model is available for this study that would help us improve the model predictability with respect to the cosmetic outcome for the patient.

The goal of this work was to progress with the agile development of both the multiscale model and the clinical study in order to build some credibility to our model, and identify the dominant phenomena that should be carefully monitored. Patients at high risk for deformation include women with relatively small breasts, with volume loss due to previous biopsies, those with high tumor-to-breast size ratio, and those with tumor positioned in specific areas of the breast—such as those immediately beneath the nipple. For this paper, we follow a single patient that seems *a priori* to offer the best possible scenario: upon initiation of the clinical study, we assessed that the cosmetic outcome for this specific patient would be very good. Therefore, we utilized this study to assess whether the impact of mass lost after lumpectomy, tissue inflammation during the healing process, and changes in stiffness of scar tissue could be quantified using our model.

We present in this paper the lessons learned from interrogating our multifactorial modeling of BCT and how we might improve this clinical study to get a broader spectrum of applicabilities to our model, while targeting improvement in the BCT procedure. In other words, this paper does not conclude on the result of a single patient study, but rather on how we can progress on the model by going from a first data set to a broader one. We advocate here that this so-called agile development of the clinical study is an optimal way to develop a predictive model, as the patient corpus grows. We will demonstrate that a multiscale model used to capture a post-surgery outcome is far more complex than one initially may think, and describe the benefit of what we have called computational surgery [14].

## Materials and Methods

### Ethics Statement

This paper reports on a single patient who went through breast conserving therapy. This represents the first case of an ongoing clinical protocol that has been approved by the Houston Methodist Research Institute institutional review board—IRB0112-0007.

After having the study explained to her and having read the study description, the patient provided informed written consent for the acquisition and publication of data for the trial.

Participants recruitment for the trial started on May 7^th^, 2012. Because the trial was considered a single-patient case study at the time of the trial start, the trial was registered on ClinicalTrial.gov (NCT02310711) on December 1^st^, 2014 after recruitment started. The authors confirm that all ongoing and related trials for this drug/intervention are registered on ClinicalTrial.gov.

The study protocol and the TREND checklist are available as supporting information; see S1 Protocol and S1 TREND Checklist.

### Data acquisition and clinical trial

Factor influencing the cosmetic outcome of BCT are multiple and include [15, 16]:

- the position and size of the lumpectomy within the breast,
- the mechanical parameters of the breast tissues,
- the distribution of fat and glandular tissues in the breast,
- the advancement of the healing process,
- the change of the mechanical properties of the tissue due to the radiotherapy treatment and/or the healing process.

**Fig 1:**
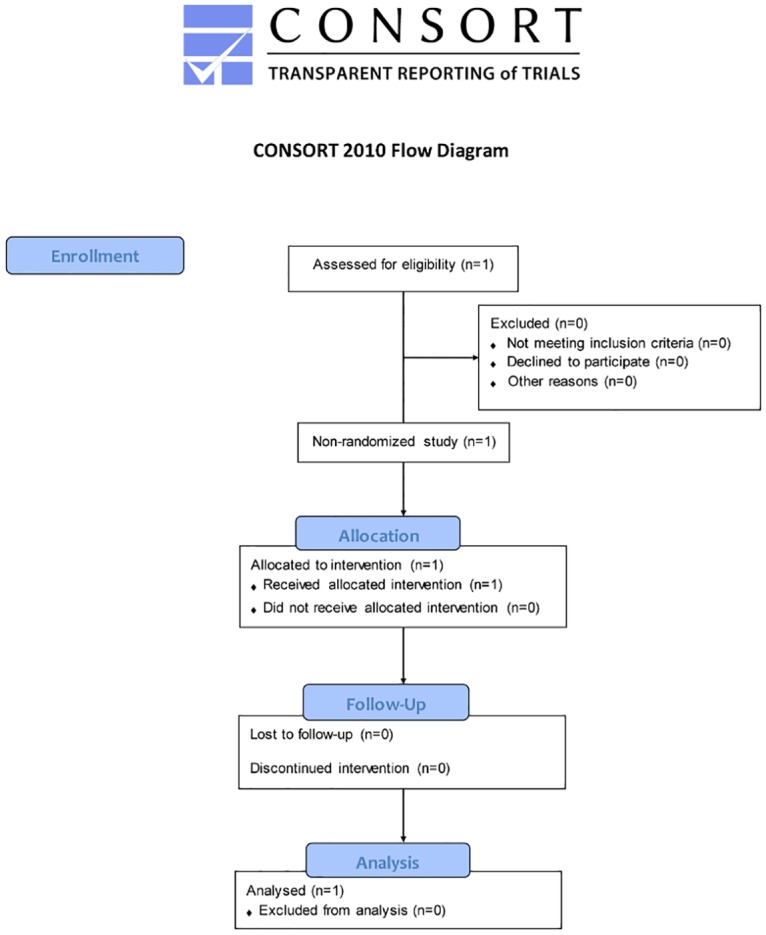
CONSORT flow diagram. Flow chart for the patient enrolled in the study.

**Fig 2:**
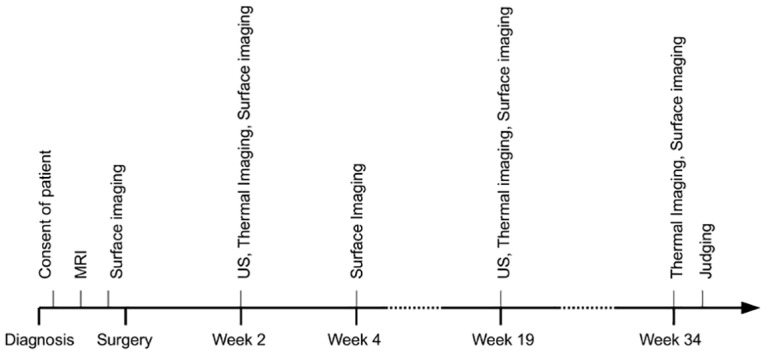
Timeline of the clinical study. Calendar of the medical exams and dates at which the data acquisition was performed during the clinical study.

The study was designed to include adult females ≥ 30 years old who had early stage non-metastatic breast cancer, received a pre-operative mammogram within 30 days of surgery, have received a pre-operative MRI exam within 30 days of surgery, would be planning to undergo lumpectomy, and receive post-surgery radiotherapy of the whole breast (Fig 1). Females who had a previous breast cancer, who required neo-adjuvant therapy, who were pregnant or breastfeeding, or who had participated in an investigational drug study in the previous 30 days were excluded. Following consent, and prior to surgery, 3-dimensional surface imaging was captured and the study began (Fig 2).

Pre-operative MRI data allowed retrieval of the 3D shape of the breast as well as the mapping of tissue distribution. Breast tissue was then classified into fat, glandular tissue or skin, and the chest muscle wall as well as the tumor were segmented. After the surgery, subsequent surface imaging provided quantitative information on the breast surface for quantification of the cosmetic defect. Observation of the possible change of coloration at some skin location, due to inflammation, necrosis or radiotherapy was accomplished using digital photography. Ultrasound imaging allowed quantification of the shape of the cavity left by the lumpectomy as healing progressed. Thermal imaging (using a FLIR E60 camera with a sensitivity of 0.1°*C* in the range of physiological temperature of interest and a spatial resolution of 240 × 180 pixels) provided an indirect indicator of the inflammation of the breast tissue. Inflammation is part of the healing process as well as a side-effect of the radiotherapy treatment on tissue [17, 18]. Finally, pain assessment was obtained at each visit.

**Fig 3:**
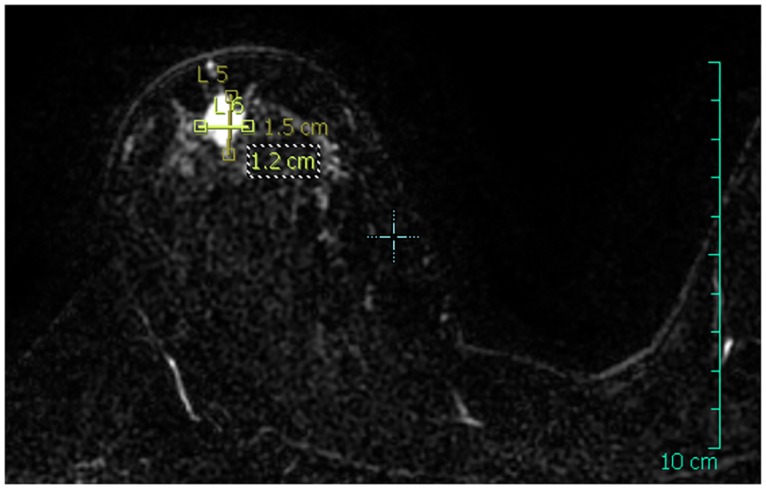
Right breast MRI in the transverse plane. The dimensions of the tumor were measured and annotated on the MRI image.

**Fig 4:**
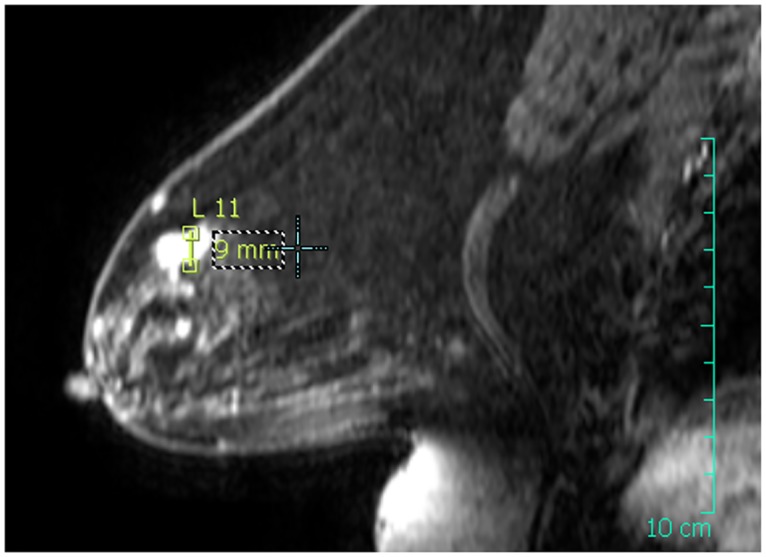
Right breast MRI in the sagittal plane. The dimensions of the tumor were measured and annotated on the MRI image.

The patient presented here is a 50-years old female with lobular carcinoma in-situ located in the right upper quandrant of the right breast, above the nipple area. The patient was monitored from June 27^th^, 2012 to February 20^th^, 2013 for a total duration of 34 weeks following surgery due to the fact that she required chemotherapy prior to radiotherapy. The week 3 study visit took place prior to chemotherapy and the week 6 study visit occurred after the course of chemotherapy. This pathology presentation was an ideal configuration with respect to size, location and development of the tumor. The tumor was localized in the sagittal plane going though the nipple with the center of the tumor at a distance of 47 mm from the skin. MRI data were acquired 30 days prior to the operation (Figs 3 and 4).

From this MRI data, we retrieved a data set composed of 199 slices with a resolution of 250 × 250 pixels and a voxel size of 0.7031 × 0.7031 × 1 mm. After the surgery, and throughout the duration of the clinical trial, the patient underwent ultrasound imaging combined with thermal surface imaging at week 2 and week 19 after surgery. An additional acquisition of thermal surface imaging was performed at week 34 after the surgery. The two-dimensional ultrasound imaging was acquired with a spatial resolution of 0.1 mm in both dimensions and was performed in the sagittal and transverse planes of the breast with the patient seated comfortably. Lateral and frontal photographs of the breast were acquired at weeks 2, 4, 19 and 34 after the surgery date as well as prior to the surgery.

Because of the tumor location centered in the sagittal plane going through the nipple, we opted for a simplification towards a 2-dimensional problem, where the transverse direction of the breast is ignored. This was one of the reasons for which we started with this specific patient, and was also in agreement with the fact we operated with a standard two-dimensional ultrasound system that could not provide an accurate 3D reconstruction of the lumpectomy cavity. The tumor was measured as a compact ellipsoid-shaped mass of 12 × 9 mm in the considered sagittal plane (Fig 4). Thanks to this 2D simplification, 3D surface imaging was not necessary and profile photographs of the breast were used to reconstruct a 2D contour in the sagittal plane going through the nipple of the right breast.

In the future, we plan to employ a more elaborate data acquisition process to validate and improve our model. However it should be noticed that, unless one shows that some essential observations are missed by current standard procedures, a predictive clinical model of cosmetic outcome that is patient-specific should not require anything but a preoperative MRI scan (or preferably a mammography), 2D ultrasound and possibly surface imaging with a low-cost system.

### Virtual breast reconstruction

**Fig 5:**
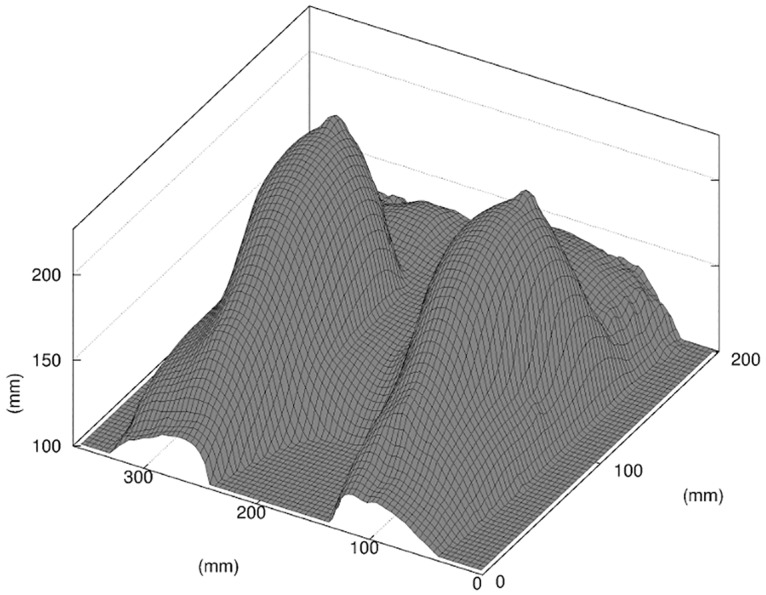
3D reconstruction of the MRI data. In this figure only the elevation map of the skin surface is shown, scaled to the MRI voxel resolution.

A 3D reconstruction of the breast surface is obtained by segmentation of the post-operative MRI data. The MRI DICOM data is pre-processed using the DICOM ToolKit (http://dcmtk.org/); the DICOM images are first converted to 8-bit monochrome images by applying a Value Of Interest windowing function computed from the histogram of each DICOM image. Each image is then processed with the Octave software (http://www.gnu.org/software/octave/); first, a segmentation using a Fuzzy C-Means algorithm is performed [19]. The data is segmented into 3 classes labeled as background noise, fat and glandular tissue. Additionally, the surface of the skin defined as the interface between the background noise and the breast tissues is smoothed using a 1D Tikhonov regularization [20] on each slice in order to correct the defects of the non-localized segmentation. The breast-chest wall interface is interpolated from a manual segmentation of the projected 3D data in the transverse and sagittal planes. Finally, the data is scaled-down to the voxel resolution of the MRI data. The resulting reconstructed surface of the skin is shown in Fig 5.

As previously discussed, we chose to work in the 2D space of the sagittal plane of the tumor bed in the right-side breast. However, and for compatibility reasons with the 3D finite elements solver used in this work, we considered a slice of thickness *δz* ≠ 0 from the reconstructed volume of the breast. In order to remove the spatial dependency in *z* required for our 2D model, we forced a translational symmetry along the *z* axis. We extracted a single “slice of interest” from the reconstructed 3D breast at the chosen coordinate and created an artificial 3D volume by extruding the slice along the *z* axis. This allowed the use of a 3D mesh as input of the finite elements solver while forcing the solution to be two-dimensional, using appropriate no-displacement boundary conditions in the *z* direction on the 2 faces of the slice. Our finite elements computational framework is in fact ready for 3D patient specific simulation [21], but as noted earlier our ultrasound imaging of the lumpectomy cavity was only 2D.

In order to account for the loss of internal tissues resulting from the lumpectomy, we created an artificial cavity in the mesh of the breast, referred to as a “virtual lumpectomy”. We modeled the virtual lumpectomy as an ellipse parameterized by major and minor axes whose dimensions were estimated from patient data, with additional input from the surgeon. First, the dimensions of the tumor bed were measured from the MRI images; then a negative margin was added, increasing the size of the cavity. The size of the negative margin was taken from the diagnostic of the surgeon, established prior to surgery. From the MRI data, we measured the major and minor axes of the cavity bed to be 12 and 9*mm*, respectively. We used a negative margin of 5 mm, as estimated by the surgeon. This construction was cross-referenced, to some extent, with the first 2D ultrasound exam that was performed after surgery, and provided some information on the cavity left by the lumpectomy.

### Multifactorial model of BCT

Over the last few years, we have developed two main classes of model to study the change in shape of the breast post-surgery.

The first class of model is based entirely on a tissue mechanical description of the breast. Immediately after surgery, it is expected that the loss of tissue left by the lumpectomy will significantly affect the breast shape. In fact, the gap left by the resection fills quickly with seroma and some pressure accumulates in this pocket due to inflammation [22]. A short-term model of the breast shape should take into account both of these phenomena.

To build this mechanical model, we built a robust model of breast tissue deformation, even before a virtual lumpectomy could be applied to the geometry. The breast is a fairly mobile structure: the MRI data is acquired in the prone position, while the cosmetic outcome is generally evaluated when the patient torso is vertical. This is a fairly nontrivial problem that has been extensively reported in the literature [10, 23–28]. We used a combination of quasi-incompressible isotropic Neo-Hookean materials to model the glandular, fat and skin tissues and the patient-specific distribution of these tissues that we were able to map from the MRI data analysis. The Neo-Hookean hyperelastic model is defined by its strain energy density function:

$$\begin{array}{c} W=\frac{\mu }{2}\left(J^{-2/3}I_{1}-3\right)+\frac{K}{2}{\left(J-1\right)}^{2}, \end{array}$$ (1)

where *W* is the strain energy per unit of volume, *I*_1_ is the first invariant of the left Cauchy-Green deformation tensor, *μ* is the initial shear modulus of the material, *K* is the bulk modulus and *J* is the determinant of the elastic deformation gradient. The parameters *μ* and *K* represent, respectively, the stiffness and compressibility for each type of tissue. In this paper we chose to use the Young modulus *E* and Poisson ratio *ν* as parameters, from which the *μ* and *K* parameters can be easily derived. This choice was motivated by the fact that those parameters are widely used in the literature and thus their range of variation can be estimated more easily.

We should mention that in the future, we will use the same hyperelastic material approximation to model the scar tissue resulting of cellular proliferation inside the wound during the healing, but we did not include that approximation in this report.

The mesh of the virtual breast was created using the Gmsh software [29] (http://www.geuz.org/gmsh/). The element sizes of the mesh are constrained to the spatial resolution of the MRI data, and a material label was assigned to each node of the mesh based on the result of the segmentation of the MRI data. The skin was modeled using triangular shell elements on the outer surface of the breast with a 1 mm thickness [24, 30]. To account for the effect of the seroma in the wound cavity after surgery, we applied a positive pressure inside the cavity. We used the finite element software FEBio (http://www.febio.org/) to solve this model. FEBio is specifically focused on solving nonlinear large deformation problems in biosolid mechanics [31].

The second class of model is a mecano-biological model that takes into account the healing process. This model is required to describe its long time scale. The cosmetic outcome may be less satisfactory after a few months of healing. It is well known that the cavity left by the lumpectomy gets filled progressively by scar tissue with a higher stiffness than the original breast tissues, and that in this long-term process up to 20% of the volume may be lost [32].

We have, therefore, developed a multiscale model that couples the modeling of the breast tissue mechanics and the progression of the wound healing inside the lumpectomy cavity. To model the healing process, we have developed both a level set approach similar to Javierre et al. [33] and an Agent-Based Model (ABM) to model the healing process. The ABM is a bottom up approach that describes the tissue remodeling at the cellular level, while the level set define as a “constitutive law” the velocity of the wound progression of the wall.

We proposed a very simple cellular automata (CA) model for this case [34] that is in some sense the simplest possible ABM one can use for healing, while giving results very similar to the level set model [12]. The CA operates on the unloaded geometry with a discrete hexagonal grid that has 3 axes of symmetry. Each site of the hexagonal grid might be either empty or occupied by a cell. The probability *p* of cell division inside the tissue, as detailed in [12], is written as:

$$\begin{array}{c} p\left(u_{i,j}\right)=F\left(c_{i,j}\right)\left(\alpha_{0}+\alpha_{1}\frac{E_{i,j}}{E_{max}}\right), \end{array}$$ (2)

where (*i*, *j*) is the site location coordinate the hexagonal grid. Cell division is promoted by a generic growth factor *c*_*i*,*j*_ modulated by a cut off function *F*, and possibly a normalized distribution of mechanical stress *E*_*i*,*j*_ in the tissues at that corresponding location in the unloaded geometry.

The equation leading the diffusion of growth factor in the tissues and developed by Javierre et al. [33] follows the equation:

$$\begin{array}{c} \frac{\partial c}{\partial t}-D\Delta c+\Lambda c=\chi_{a.l.}, \end{array}$$ (3)

where *D* and Λ are the diffusion coefficient and decay rates, respectively. *χ*_*a*.*l*._ is a unit step function equal to 1 inside a layer of fixed witdh around the wound edge, referred to as an “active layer”, and 0 elsewhere. We used homogeneous Neuman boundary conditions at the wall of the resection to express that there was no flux of growth factor. We completed the PDE problem with a homogeneous Dirichlet boundary condition on the far field that did not impact the growth factor distribution in the active layer.

The mechanical code follows the hyperelastic model described above. We then alternated between the calculation of the “instantaneous” mechanical deformation of the breast for a given geometry and the wound healing progression. The CA model for wound healing was run for a period long enough to observe a noticeable change in the closing of the wound that would change the output of the mechanical model. Typically we run the CA to model a physical period of the order of a week.

**Fig 6:**
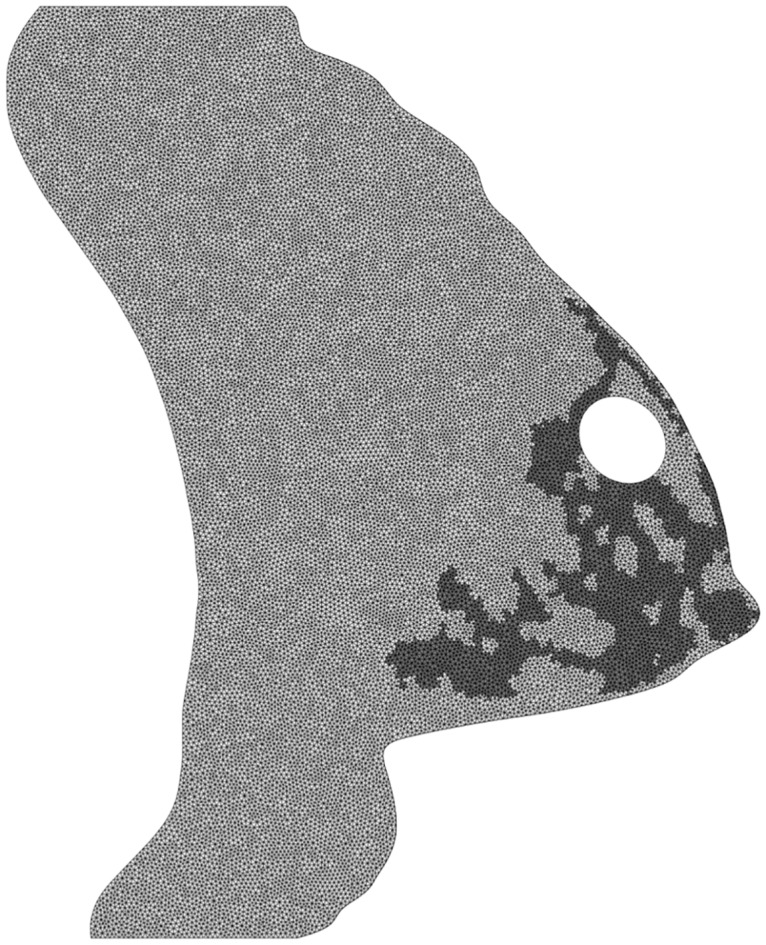
Mesh of the virtual breast, in the sagittal plane of interest. We show in a different color intensity the fat and glandular materials of the elements of the mesh.

**Fig 7:**
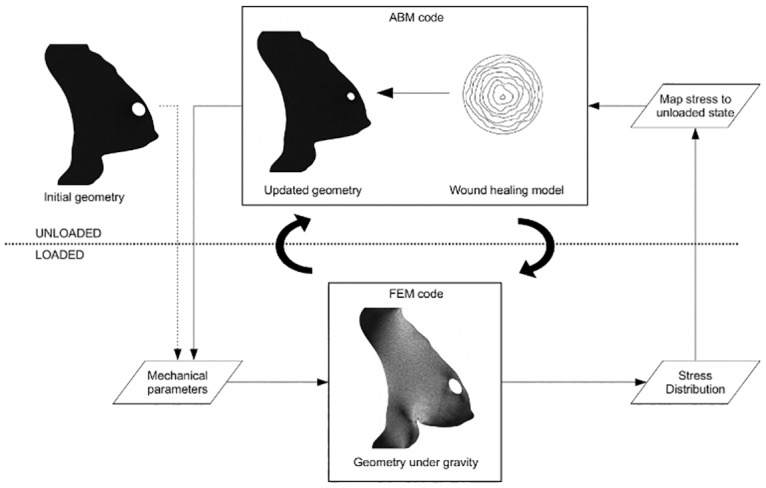
Illustration of the mechanico-biological model of the breast after BCT. Upper half of the diagram (*unloaded*): biological agent-based model of the wound healing; lower half of the diagram (*loaded*): mechanical model of the breast. The stress distribution at the wound edge is normalized and mapped from the loaded geometry of the breast under gravity to the unloaded geometry of the breast of the biological model.

The virtual breast was then updated with the new geometry of the wound. A new mesh of the virtual breast was created as shown in Fig 6. A detailed description of the implementation of that multiscale model from Garbey et al. [12] is summarized in Fig 7.

Once the healing was completed by the mecano-biological model, we returned to the initial mechanical model, but using a stiffer material for the newly-formed scar tissue, according to the literature. Next, we calibrated the model.

### Data fitting and model calibration

Table 1 summarizes the parameters set for each component of the model, mechanical or biological. Some parameters such as density or compressibility of soft tissue can be set relatively easily from the literature. Other parameters such as *α*_1_ that accounts for the sensitivity of wound healing to mechanical stress have a secondary effect in the simulation. We set *α*_1_ = 0, justified by that fact that its effect in the simulation would be negligible versus the error resulting of the data acquisition process. We also used a generic growth factor *c*, but we have no lab measurement available that would help us specify its decay rate Λ. However, by using a production rate overestimated to *χ*_*a*.*l*._ = 1 [*c*].*s* ^−1^ inside the active layer, we significantly lessen the influence of Λ. It turns out in our simulation that this parameters has also secondary effect that would be less significant than the error we have made on the wound cavity location. Eventually, we can restrict ourselves to a fairly limited set of unknown parameters (see Table 2).

**Table 1:** Summary of the parameters of the model (i).

**Parameter**	**Value**
Mechanical model of breast tissues:	
*ν*_*fat*_	0.49
*ρ*_*fat*_	0.9 kg.L^−1^ [45]
*ν*_*glandular*_	0.49
*ρ*_*glandular*_	1 kg.L^−1^ [46]
*ν*_*skin*_	0.49
*ρ*_*skin*_	1 kg.L^−1^
*δ*_*skin*_	1 mm [47]
First phase of healing:	
*E*_*inflammation*_	80 kPa [43]
*δ*_*inflammation*_	5 mm
Third phase of healing:	
*ν*_*scar*_	0.49
*ρ*_*scar*_	1 kg.L^−1^
Biological model of wound healing:	
*D*	0.05 cm^2^day^−1^ [48]
Λ	0
*α*_1_	0

These parameters are extracted from the literature or set to zero if they appears to have secondary effect in the model sensitivity to parameters.

**Table 2:** Summary of the parameters of the model (ii).

**Parameter**	**Value**
Mechanical model of breast tissues:	
***E***_***fat***_	2–15 kPa [49]
***E***_***glandular***_	5–15 kPa [49]
***E***_***skin***_	5–30 kPa [50]
First phase of healing:	
**λ**_**1**_	1.0–3.0
***P***	0.0–500.0 Pa
Second phase of healing:	
**λ**_**2**_	1.0–3.0
Third phase of healing:	
**λ**_**3**_	1.0–3.0
***E***_***scar***_	10–80 kPa
Biological model of wound healing:	
***α***_**0**_	0–0.6

The parameters in bold characters are used to define the sets of unknown parameters *α* estimated in this paper. Initial ranges of variation are given here as used in the optimization method.

We have put in bold characters the set of candidate unknown parameters that we would like to be patient specific. These are the few parameters that we intend to recover from model fitting. Let us denote *α* this unknown vector of scalar. Since we worked with a 2D model with this first patient, our criteria to match the model with the experiments were extremely simple. We looked at the difference between the 1D contour of the profile view of the patient’s breast and the same profile obtained by the simulation.

We used standard photographs of the patient side view obtained during clinical examination. We extracted the contour of the breast easily with a segmentation method. Let us call *C* ^*ground*–*truth*^ the corresponding contour. Meanwhile let us denote *C* ^*model*^ the same supposedly contour, obtained from the simulation. *C* ^*ground*–*truth*^ and *C* ^*model*^ are parametric curves. We projected both function on a square ROI that included the breast on a discretized grid that corresponded to the MRI resolution at the millimeter scale. The digital pictures were less accurate and thus required interpolation.

The objective function *f*(*α*, *s*) we minimized is defined as:

$$\begin{array}{c} f\left(\alpha ,s\right)=\sum_{i,j}\|C_{i,j}^{model}\left(\alpha \right)-s*C_{i,j}^{ground-truth}\|. \end{array}$$ (4)

We did not require accurate measurement of the distance from the patient to the camera, in order to simplify the clinical data acquisition process for the nurse and save time for the patient. The physical dimension of the pixel in the image expressed by the aspect ratio *s* was then set as an additional unknown in the optimization procedure.

Before we proceeded with the parameter estimation, we made some preliminary observations in the clinical study with the first patient to determine which phenomena seemed to emerge clearly and therefore what model might be able to catch it. We will present some simple metrics we can extract from the imaging of this patient.

## Highlighting the three phases of healing

**Fig 8:**
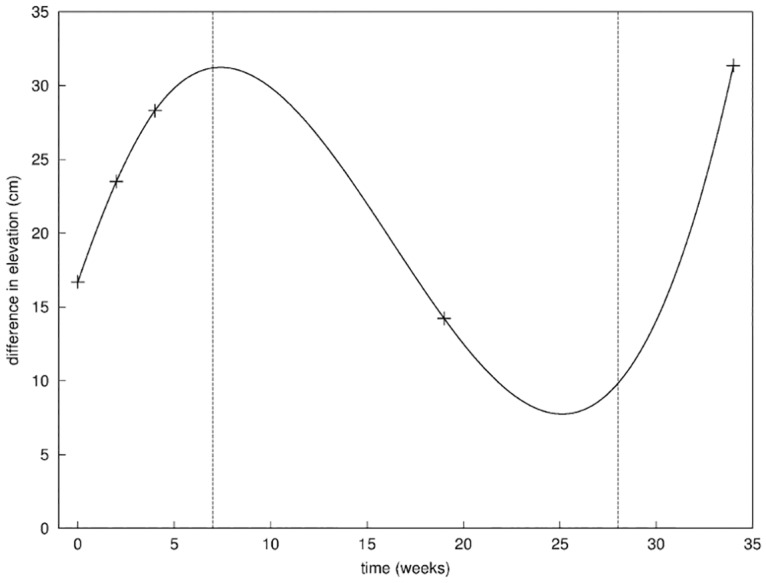
First metric. Evolution of the difference in the vertical position of the nipple of the right breast and the left breast; the three phases of healing are separated by the vertical dashed lines.

From the series of frontal photographs of the patient during the clinical protocol of Fig 2, we notice that the difference in height between the left and right nipple changed over time in a fairly non trivial way. We defined the first metric as the absolute vertical difference in height between the nipples of each breast of the patient (Fig 8). We tracked the potential change of tissue stiffness that could affect that metric.

**Fig 9:**
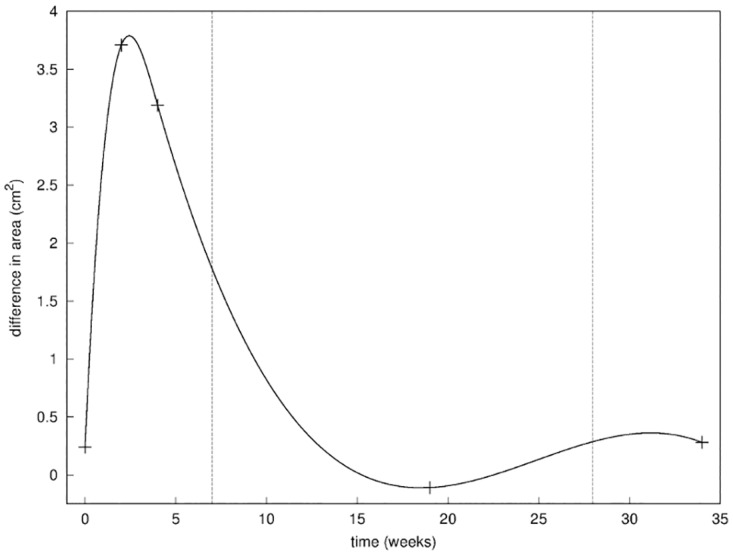
Second metric. Evolution of the difference in the lateral surface of the right breast and the left breast; the three phases of healing are separated by the vertical dashed lines.

Similarly, using the lateral photographs of the patient, we were able to compute the area of the breast included in a disc of fixed radius centered on the nipple, and compared the area of both breasts. This complemented the first metric, giving an estimate of the change of volume of the operated breast (Fig 9). We tracked potential change due to the internal loss of tissue after the surgery as well as the creation of scar tissues inside the wound, counterbalanced by the change of volume due to the accumulation of seroma.

The curve obtained with the first metric (Fig 8) highlights the 3 distinct phases of healing [35, 36] that we will investigate with our model later on in this paper. In the first seven weeks after the operation, the progressive increase of the difference in elevation of the breasts from 17 to 28 mm indicated an increase of stiffness, possibly explained by the inflammation of the wounded tissues [36]. The following decrease of the difference in elevation to about 14 mm indicates a return to a relaxed position of the breast over the next 4 to 5 months; this was interpreted as the diminution of the inflammation phase coupled with the progress of cellular division and tissue remodeling inside the wound [37]. Finally, the resumption of the difference in elevation around the 8^th^ month to 32 mm indicated an increase of tissue stiffness due to the radiotherapy [38] that the patient underwent in the scope of the breast conserving therapy.

Those results were validated by the second metric (Fig 9) showing the evolution of the difference in lateral area, that we assimilated to the volume of the breast. The first two phases of healing and their time scale were clearly identified. The inflammatory phase was characterized by a large augmentation of the lateral area of 3.5 cm^2^ due to the pressure resulting from the accumulation of seroma [39, 40], filling the cavity during the first few weeks of healing and typical of the post-lumpectomy inflammation. This was followed by return to a normal configuration where the two breasts are roughly of the same lateral area. This can be explained by the fact that the internal pressure resorbs itself while the scar tissue created during the 2^nd^ phase of healing fills the cavity.

**Fig 10:**
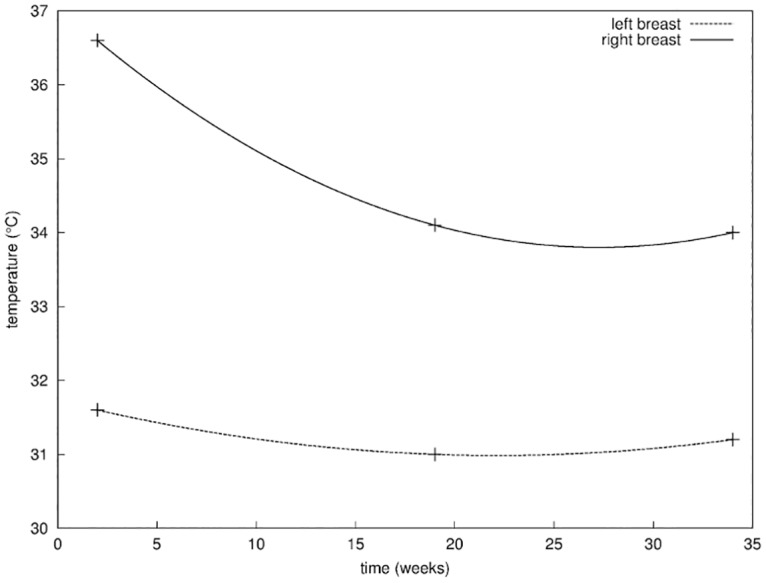
Third metric. Evolution of the surface temperature of both breasts, measured at the location of the surgical incision on the skin on the right breast, and the corresponding location on the left breast.

Finally in Fig 10 we report on the evolution of the difference in temperature between the two breasts measured from thermal imaging. The surface temperature of the right breast was measured at the location of the surgical incision on the skin. The surface temperature of the left breast was measured at the corresponding location on the left breast. The temperature was measured on a grid of 7 × 7 pixels and averaged in order to minimize the signal-to-noise ratio. The difference in temperature appears initially higher of about 5°*C*, confirming the inflammatory activity in the operated breast [41]. During the healing process, a convergence in the temperatures of the two breasts was observed, indicating a decay of the inflammatory activity.

In conclusion we have identified three phases of the healing process:

- Phase I: weeks 1–6, characterized mainly by post-surgery inflammation.
- Phase II: weeks 7–20, characterized by seroma absorption and progression of the healing.
- Phase III: weeks 21–35, dominated by the effect of radiotherapy.

The patient status at week 0, *i.e.* right before surgery, is referred to as Phase 0.

The 3 measurements presented in this section were easy to do. Perhaps we should have seen the patient more often to fill our potential measurement gap around weeks 10 and 25 in Fig 8. Beside, this scenario would have changed somehow if the patient had not got any positive lymph node after surgery. The radiotherapy treatment started here later than usual because of the chemotherapy treatment from week 6 to 16. Nevertheless, we could adapt our general computational framework to describe each phase and recover a subset of the patient specific parameters in our model.

## Results

**Fig 11:**
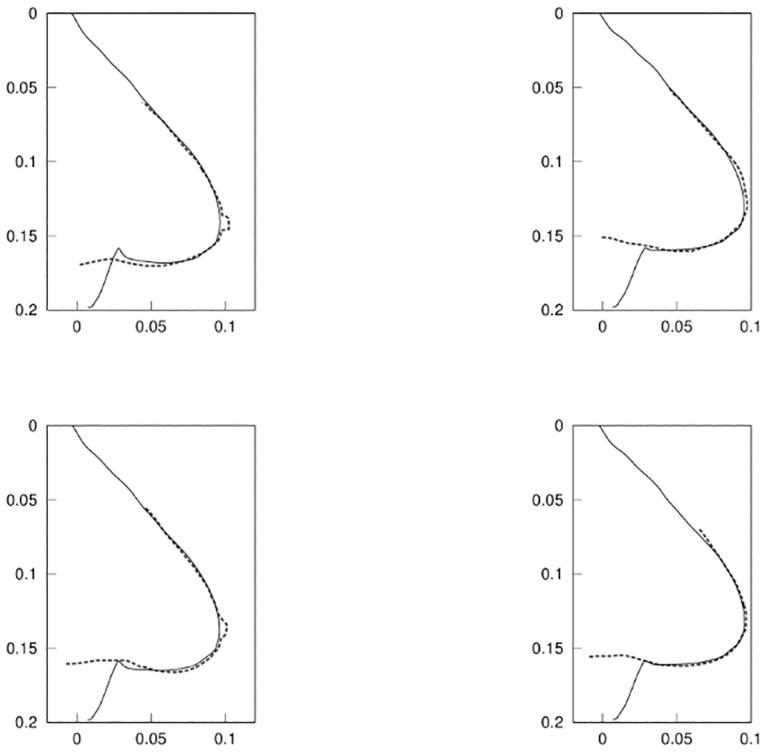
Fitting of the model to the patient data for the different phases of healing. Result of the 2D fitting of the parameters described in Table 2 for Phase 0 (estimation of the mechanical parameters *E*_*fat*_, *E*_*glandular*_, *E*_*skin*_ before surgery, top-left quadrant), Phase I (fitting of the parameters λ_1_, *P* for the inflammation of the breast, top-right quadrant), Phase II (fitting of the biological parameter *α*_0_, bottom-left quadrant), and Phase III (fitting of the parameters λ_3_, *E*_*scar*_ for the increase in stiffness of the breast during the radiation therapy, bottom-right quadrant). Solid lines represent the output of the model *C* ^*model*^ and dashed lines represent the breast contoured manually *C* ^*ground*–*truth*^ (axes in meters).

In this study, we have limited ourselves to a few parameters for the validation of each phase of healing to get a meaningful and credible result. In fact, the more unknown parameters we can work with, the better fit we should get. With only two parameters, we are able to show graphically the parameter landscape with a surface response [42] and improve our understanding of the phenomenon post-surgery. We will also demonstrate that it is feasible to have an accurate prediction in each phase (Fig 11).

**Phase 0:** First, we estimated the Young modulus of fat, glandular and skin tissue of the patient that fitted to the breast contour acquired in the seated position. For this, we used *α* = {*E*_*fat*_, *E*_*glandular*_, *E*_*skin*_} in Eq (4).

We assumed that the patient’s breast in the prone position during the MRI scan had no residual stress. We needed to somehow invert the gravity before applying the model to the seated position with vertical gravity. We have previously described this procedure [10, 11]. Technically, one can improve this method and skip that computational step by using a floating device during MRI acquisition [27] in order to directly acquire the unloaded shape.

**Fig 12:**
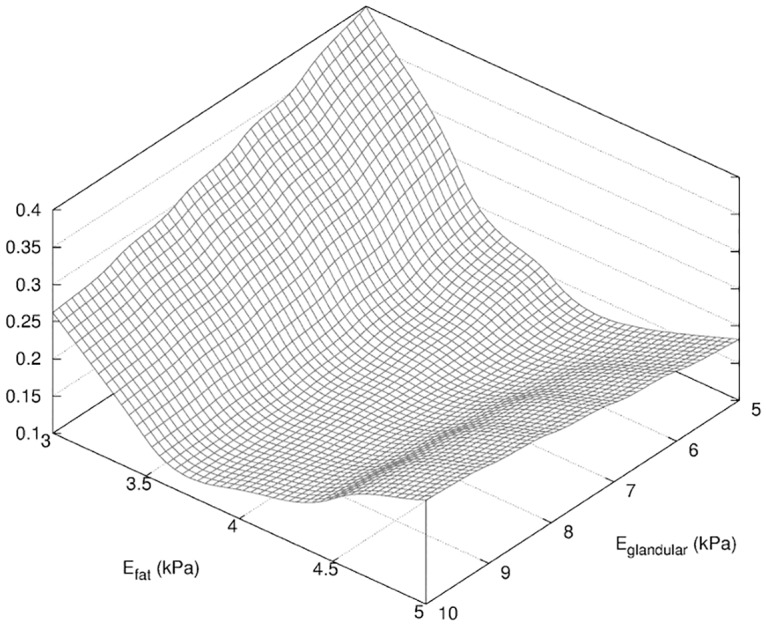
Surface response of *f* for Phase 0. Surface response of *f* (see Eq 4) obtained in the determination of the mechanical parameters *E*_*fat*_, *E*_*glandular*_, *E*_*skin*_ described in Table 2.

We obtained a minimum of the objective function *f* for *E*_*fat*_ = 3.7 kPa, *E*_*glandular*_ = 9.5 kPa and *E*_*skin*_ = 25 kPa. We observed in the surface response shown in Fig 12 a higher sensitivity to the Young modulus of the fat tissue. This could be a consequence of the fat tissues occupying the majority of the breast, as compared to the skin and glandular tissues more scarcely distributed.

Once the mechanical properties of the breast were established, we attempted to model each phase of the healing process.

**Phase I:** In the first phase of healing, we observed that the inflammation of the breast induced an increase of volume by an order of magnitude of the size of the resected tissue volume. We also observed a higher elevation of the nipple. Those observations might be associated to two distinct phenomena characteristic of the inflammation process; on one hand the pressure resulting from the seroma filling the wound cavity raised during the first weeks of healing. On the other hand, we may have observed an increase of the stiffness of the breast tissues.

To model the first effect of the inflammation we applied a normal positive pressure *P* on the walls of the cavity. To model the second effect of the inflammation, we defined a new Young modulus $E_{fat}^{\prime }=\lambda_{1}E_{fat}^{0}$ and $E_{glandular}^{\prime }=\lambda_{1}E_{glandular}^{0}$ for the fat and glandular tissues, respectively, where λ_1_ is an unknown scalar. To minimize the number of unknown parameters, we consider the effect of the swelling as homogeneous with respect to the fat and glandular tissue stiffness. We can use then a unique scaling factor λ_1_ for tissue stiffness that applies to both fat and glandular tissues. The skin Young modulus is unchanged from Phase 0 as as the effects of inflammation are more prevalent on internal soft tissues. We assumed a region of constant width *δ*_*inflammation*_ = 5 mm around the wound edge modeling the local inflammation. We modeled this region of higher stiffness with a Young modulus of *E*_*inflammation*_ = 80 kPa [43]. We also assumed that healing at this early phase had not enough time to produce new scar tissue.

**Fig 13:**
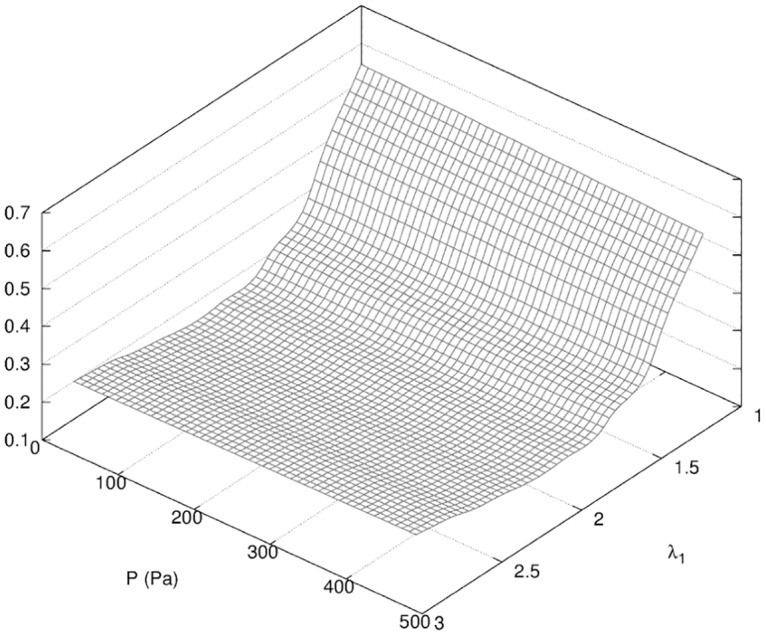
Surface response of *f* for Phase I. Surface response of *f* (see Eq 4) obtained in the determination of the parameters λ_1_, *P* described in Table 2.

**Fig 14:**
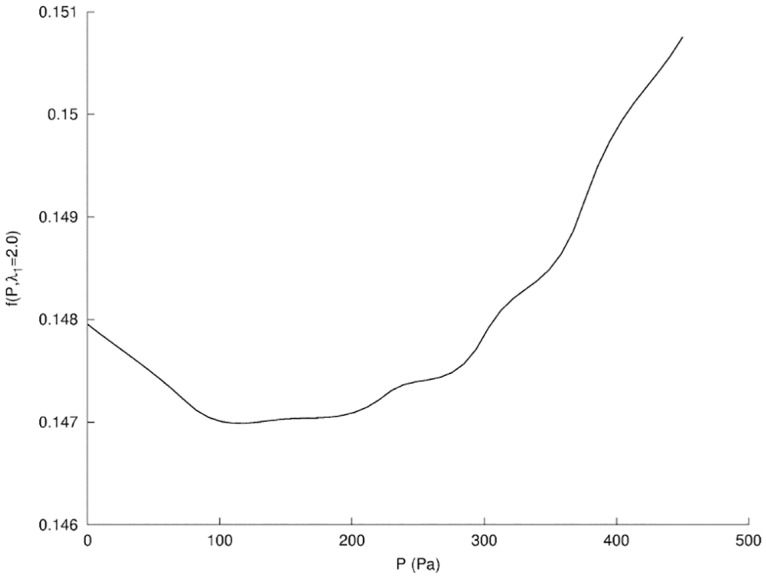
Projection of the surface response of *f* for Phase I. Projection of *f* for Phase I (see Fig 13) in the plane λ_1_ = 2.0.

The new vector of unknown in Eq (4) is *α* = {λ_1_, *P*}. We obtained a global minimum of our objective function *f* that led to the best fitting in Fig 11 for *P* = 120 Pa and λ_1_ = 2.0. The surface response in Fig 13 indicated a much higher sensitivity in the dimension of the parameter λ_1_, where the fitting appeared nearly independent from the pressure *P*. We explain this result by the fact that the filling of the wound cavity by seroma increased *locally* the volume of the breast at the location of the wound while Eq (4) provided a global metric of the breast contour. The dependency in *P* is shown in Fig 14 where the global minimum is visible.

While the resulting fitting using a single scaling factor λ_1_ was remarkably good, we may consider an independent increase in stiffness for the fat and glandular tissues in the 3D case. As a matter of fact, the 3D fitting of our model might be more difficult to achieve than in the 2D case, but will also provide more data to retrieve additional parameters. The model fitting then confirmed a doubling of the stiffness of the breast tissues and a positive pressure in the cavity left by the resection. Both are necessary to get the best fitting. The global increase of tissue stiffness seemed, however, to be prevalent.

We noticed in Figs 8 and 9 that the effect of seroma increasing the pressure in the cavity and the increased stiffness of the tissue was somehow reversible. As a matter of fact we assumed that no healing had progressed yet. In other words, once the excess of seroma was managed by the lymphatic network, and the tissue stiffness relaxed to a lower value, the tissue shape should have no memory in the time scale of a week. We then restarted the simulation of the breast tissue deformation with the mechanical parameters corresponding to the relaxed state of Phase 0.

As a result of this quasi-steady assumption, we assumed for the next phase that the pressure in the cavity would return to zero, and that the dominant effect was dependent on the change of geometry of the cavity that became smaller.

**Phase II:** In the second phase of healing following the inflammation, the increase in production of growth factors such as PDGF and TGF-*β* triggers cellular activity and collagen deposition in the wound cavity [33, 44]. In this phase, we did not distinguish between the proliferative and remodeling phases of the wound healing, which mainly differ at the cellular level. At the end of the inflammatory phase, the shape of the breast may have returned to its shape at Phase 0, except that a cosmetic defect may result from the new shape of the empty cavity surrounded by newly formed scar tissues.

To take into account the progress of the healing of the wound, we used our coupled mecano-biology model for the duration of 19 weeks corresponding to the next clinical exam with our patient. The cell division probability *p* is correlated to the velocity at which the wound edge progresses in its normal direction with the parameters *α*_0_, *α*_1_. We neglected the secondary effect related to the mechanical stress by setting *α*_1_ = 0 as justified in Section 1; we restricted our search for *α*_0_ in the parameter range [0, 0.6]. We also included a uniform multiplicative factor λ_2_ to account for the increase in stiffness of the newly created scar tissue as well as the surrounding tissues in the breast while retaining the hypothesis introduced in Phase 1.

The new vector of unknown in Eq (4) is *α* = {λ_2_, *α*_0_}. We obtain a global minimum of the objective function *f* for λ_2_ = 1.2 and *α*_0_ = 0.17. We then had an increase of the tissue stiffness of the order of 20%. This value was significantly lower than observed for the previous phase of the healing. This confirmed our initial guess of different phases of healing characterized in part by the changes in the stiffness of the breast tissues.

**Fig 15:**
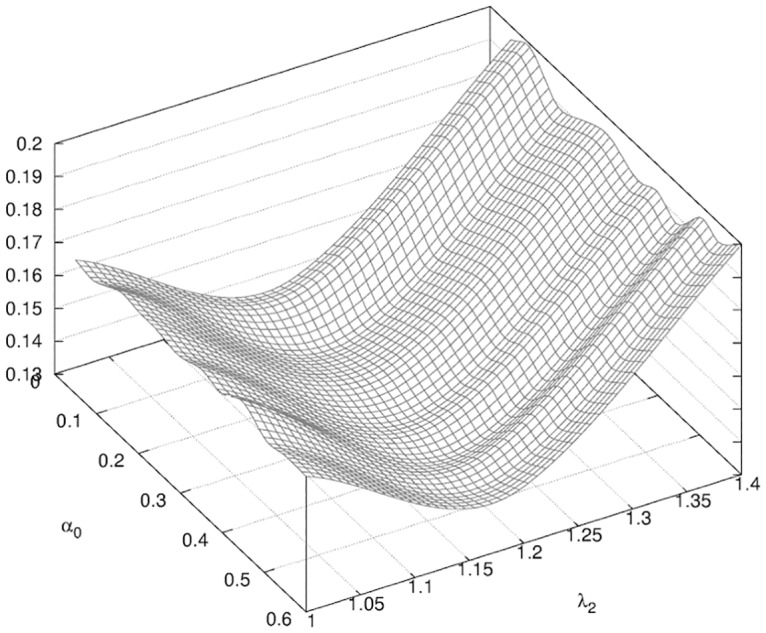
Surface response of *f* for Phase II. Surface response of *f* (see Eq 4) obtained in the determination of the parameters λ_2_, *α*_0_ described in Table 2.

**Fig 16:**
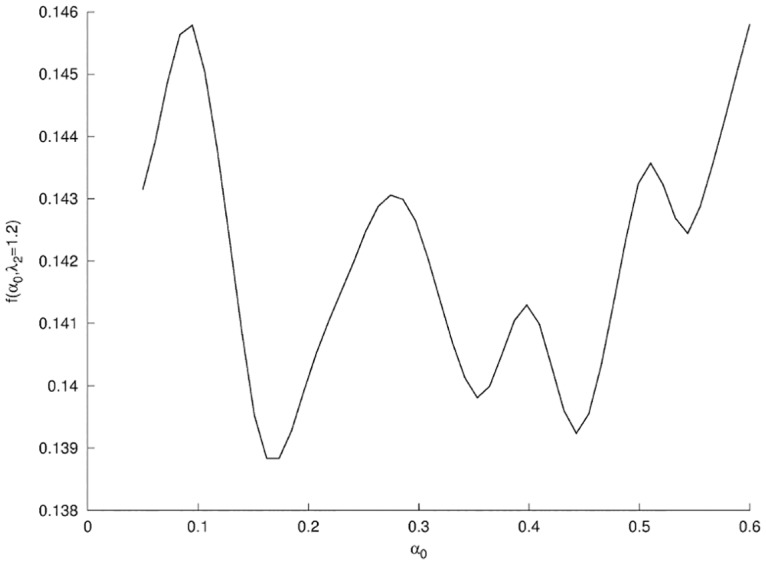
Projection of the surface response of *f* for Phase II. Projection of *f* for Phase II (see Fig 15) in the plane λ_2_ = 1.2.

The surface response in Fig 15 a higher sensitivity on parameter λ_2_ while variations due to *α*_0_ are of order of magnitude of 10% lower. When we project the surface response in the λ_2_ direction (see Fig 16) we observe multiple local minima for *α*_0_ ∈ [0.15, 0.5]; this lack of selection on *α*_0_ did not permit us to conclude quantitatively on the validation of the parameters of the biological model of wound healing.

**Fig 17:**
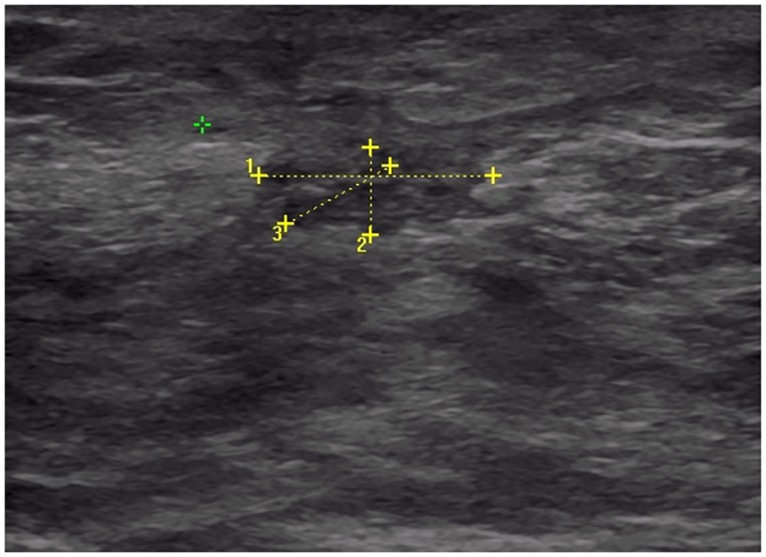
Ultrasound imaging of the wound. Image acquired in the transverse plane of the wound at week 19 after surgery. The segments 1 and 2 were measured at 13 mm and 4 mm, respectively.

With the minimum *α*_0_ = 0.17, appearing to be only marginally a global minimum, our mechano-biological model predicted, for week 19 after surgery, a cavity of size 13 mm × 8 mm in the vertical loaded position. This is actually somehow coherent with Fig 17 an ultrasound image of the patient’s breast acquired at week 19 after surgery in which the size of the cavity measured was of the order of 12 mm × 4 mm. In this context, and because of the uncertainties on the ultrasound imaging along with the small size of the wound cavity of the patient, we cannot validate quantitatively the effects of the parameter *α*_1_ just on the 2D contour of the breast. We may however be able to do better as we develop further our work using 3D data. Nevertheless, we observe in the ultrasound imaging of the wound in Fig 17 a more elongated shape of the wound cavity than predicted by our model in the absence of *alpha*_1_, consistent with the *qualitative* prediction of our model regarding the effect of mechanical stress on wound healing when *α*_1_ ≠ 0 [12].

**Phase III:** The third and last phase of the healing we have monitored seemed dominated by the impact of the radiation therapy. In general, patients undergo radiation therapy usually 2 to 3 weeks after surgery in order to minimize the risk of tumor recurrence. However our patient had chemotherapy in addition to BCT, which therefore delayed the course of radiotherapy. The patient was given external beam radiation therapy for 7 weeks, starting on week 20.

Our next and last clinical exam used to fit the model for this phase was at week 34, about 7 weeks after the end of the radiotherapy treatment. In our prediction with the mechano-biology model, the size of the lumpectomy would have been less than a centimeter at week 34. Beside, the effect of the increased curvature of the wound becomes smaller should speed up the closure [13, 33]. We assumed then that the wound was closed at week 34.

In this phase, we introduced in the mechanical model the newly created scar tissue filling the lumpectomy cavity with a Young modulus *E*_*scar*_ unknown *a priori*. Tissues density and Poisson ratio are supposed to be uniform across the breast and unchanged. We again used a multiplicative factor λ_3_ applied to the fat and glandular tissues in order to model the stiffness increase caused by the radiation treatment.

**Fig 18:**
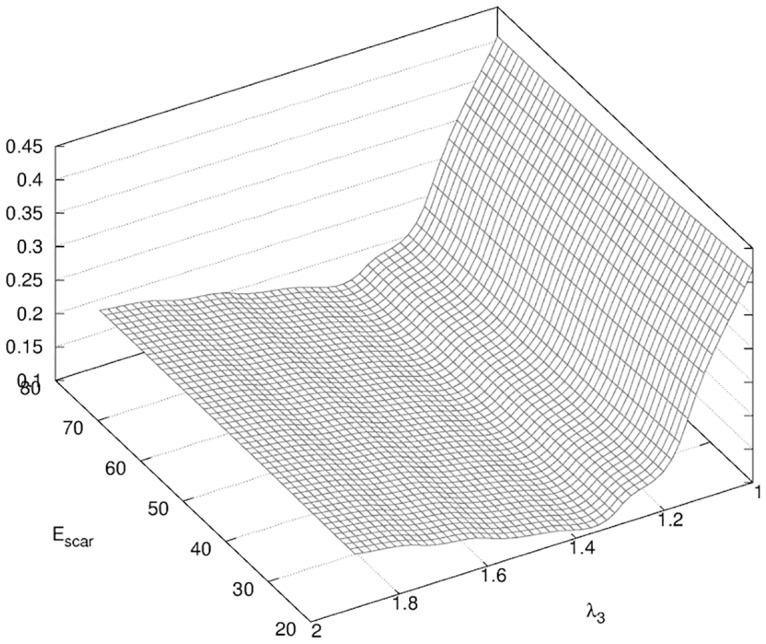
Surface response of *f* for Phase III. Surface response of *f* (see Eq 4) obtained in the determination of the parameters of the model λ_3_, *E*_*scar*_ described in Table 2.

The new vector of unknown in Eq (4) is *α* = {λ_3_, *E*_*scar*_}. We obtained a minimum of the objective function *f* shown in Fig 18 for *E*_*scar*_ = 24 kPa and λ_3_ = 1.7. This increase in tissue stiffness from the second phase of healing was in accordance with our observation of the patient and confirmed our initial estimate of the presence of three distinct phases of healing. However, the very small variations of the surface response of *f* in the dimension of *E*_*scar*_ implied that our prediction on the Young modulus of the scar tissues was not really conclusive.

## Discussion

We have begun with the first patient enrolled in our clinical study to interrogate a multifactorial model of breast recovery after surgery. The goal was to confirm our heuristic knowledge on the phenomenology. We have been able to confirm with this work that BCT outcome is indeed multiscale by nature. Thanks to our general mechano-biological model framework, we are able to study separately the effects of each factor on the breast shape such as change of stiffness in tissues, positive pressure in the wound cavity, production of scar tissue, etc.

We took a step further here and tried to retrieve the dominant factors affecting the breast shape of the patient based on clinical measurable data that can be easily incorporated in the standard health care follow up procedure. The answer is indeed time-dependent since inflammation can produce transient phenomena that temporarily mask the final cosmetic outcome.

The choice of our first patient was rather critical to start this study in close to ideal conditions: the cosmetic outcome was rather good and the location of the tumor was such that we could work quickly with 2D prediction in the sagittal plane. What we could not easily anticipate was the fact that this patient would require chemotherapy, therefore delaying radiotherapy. This resulted in some healing during the chemotherapy that we could not measure.

This study allowed us to obtain a fitting between our model and the clinical data for this patient better than what we would have expected initially. This result might be significant because we operate the fitting with a very minimum number of parameters, 2 or 3 at most, that are patient-specific. Furthermore, we have learned from this study how to improve our clinical protocol. For example, it is clear that we should see the patient more often, for a longer period of time and during any treatment phase (chemotherapy and/or radiotherapy).

This study also further justifies validation by 3D accurate reconstruction of the cavity left by the resection of the tumor with 3D ultrasound imaging. 3D ultrasound is becoming more common and affordable. Fortunately, we have acquired a 3D surface imaging capable to complete the study.

We are now pursuing this clinical study with new patients [21], and are planning for a small subpopulation to undergo MRI exams in order to obtain some ground truth measurements. We are also confident that we can pursue this study with patients who present more challenging BCT conditions. In particular, we will begin to follow up cases where tumors are at locations that may lead to dissymmetry. In this case, we will indeed use 3D modeling and perhaps get from that additional dimension in our model some improvement in our understanding of the phenomenon. Ideally, we should gradually increase the complexity of the biological component of our model and its relationship with radiotherapy. This may give us a better way to predict and possibly minimize the cosmetic outcome of BCT while controlling at best tumor recurrence.

## Supporting Information

S1 Protocol Study protocol. (PDF)

S1 TREND Checklist Transparent Reporting of Evaluations with Nonrandomized Designs (TREND) checklist. (PDF)
