# Supplementary material for: Interrogating a Multifactorial Model of Breast Conserving Therapy with Clinical Data
Source: PLoS One. 2015 Apr 23;10(4):e0125006. doi: 10.1371/journal.pone.0125006 (PMC4408022; doi:10.1371/journal.pone.0125006)
Supplement: S1 Protocol — (PDF) [file pone.0125006.s001.pdf]

C L I N I C A L   P R O T O C O L

**TITLE:**	**A Pilot Study for the Identification of Targets for Clinical Improvement in Breast Conserving Therapy**
**ORGINIAL PROTOCOL DATE:**	05 JAN 2012
**PRINCIPAL INVESTIGATOR:**	Barbara L Bass, MD, FACS<br>The Methodist Hospital<br>6550 Fannin St., SM1661<br>Houston, TX 77030
**INVESTIGATOR SIGNATURE:**	
	Barbara L Bass, MD, FACS Date
**PROTOCOL AUTHOR:**	Linda W Moore, Barbara L Bass, and Marc Garbey

This clinical research protocol will be conducted in accordance with Good Clinical Practice and the guidelines of The Methodist Hospital Research Institute Institutional Review Board.

L I S T O F I N V E S T I G A T O R S

**Principal Investigator:**	**Barbara L Bass, MD, FACS**<br>**Department of Surgery**
**Co-investigators:**	Marc Garbey, PhD
	Linda W Moore, MS<br>Department of Surgery

LIST OF ABBREVIATIONS

Term	Definition
3-D SI	3-dimensional 180° camera surface imaging
AE	Adverse Event
NCI	National Cancer Institute
TMHRI	The Methodist Hospital Research Institute

STUDY SYNOPSIS

**SPONSOR:**	The Methodist Hospital Department of Surgery
**TITLE:**	A Pilot Study for the Identification of Targets for Clinical Improvement in Breast Conserving Therapy
**OBJECTIVES:**	The primary objective of this study is<br>• to compare the surgical lumpectomy, lumpectomy healing process and surface contour changes to the predicted multiscale model to characterize<br>Secondary objectives include:<br>• provide information regarding the use of 3-dimensional surface imaging to detect changes over time in the skin surface and dimension related to lumpectomy healing and breast contour<br>• provide information on the use of ultrasound of the lumpectomy bed and changes that occur during the course of breast cancer treatment and healing<br>• to describe the course of pain experienced by women undergoing BCT and the correlation of pain to the healing process.<br>• provide information on the use of thermal Imaging to capture changes during healing
**TRIAL DESIGN:**	The proposed study is a single-center, prospective, pilot, observation study to tabulate and measure the changes that occur during treatment and healing for breast conserving therapy (BCT) in women with non-metastatic breast cancer for comparison to the multiscale model of breast lumpectomy and healing in order to identify targets for improving BCT.
**TYPE AND NUMBER OF SUBJECTS:**	The study will be comprised of 12 de novo breast cancer patients with non-metastatic breast cancer undergoing BCT.
**Main Inclusion criteria**	• Adult females ≥ age 30 years<br>• Have early stage (Stage I, II), non-metastatic breast cancer<br>• Planned to receive BCT<br>• Have received a pre-operative mammogram within 30 days of surgery<br>• Have received pre-operative magnetic resonance imaging (MRI) within 30 days of surgery<br>• Planned to receive post-surgery radiotherapy, by whole breast radiotherapy

**Main Exclusion criteria**	• Adult females younger than 30 years of age<br>• Previous breast cancer<br>• Neo-adjuvant therapy for breast cancer<br>• Participation in a study of investigational drug in the previous 30 days
**TRIAL AND TREATMENT DURATION:**	Subjects will be enrolled in the study during the 3 weeks prior to receiving their BCT surgery and will be followed for six months after the BCT surgery. A total of 6 visits (not including the surgery) are planned.

TABLE OF CONTENTS

1 BACKGROUND AND INTRODUCTION 8
1.1 APPROACH TO THE PROBLEM 9
1.2 RATIONALE FOR THE CLINICAL STUDY 9
2 PURPOSE AND OBJECTIVES 10
2.1 PURPOSE 10
2.2 OBJECTIVES 10
2.2.1 Primary objective 10
2.2.2 Secondary objective 10
3 DESIGN RATIONALE 10
4 EXPERIMENTAL PLAN 11
4.1 STUDY DESIGN 11
4.1.1 Study endpoints 11
4.1.2 Number of subjects 11
4.1.3 Study duration 11
4.1.4 Study population 12
5 TREATMENT PROCEDURES 12
5.1 OTHER CONCOMITANT STANDARD OF CARE TREATMENT 12
6 STUDY PROCEDURES 12
6.1 CONSENT PROCESS 12
6.2 ASSESSMENT OF ELIGIBILITY 13
6.3 ENROLLMENT 13
6.4 3-DIMENSIONAL SURFACE IMAGING OF THE BREAST 13
6.5 BREAST ULTRASOUND 13
6.6 PAIN SCALE SCORE 13
6.7 THERMAL IMAGING OF THE BREAST 13
6.8 JUDGING 13
6.8.1 Sequence of events 14
6.8.2 Screening and eligibility 14
6.8.3 Study completion 14
7 STATISTICAL ANALYSIS PLAN 14
7.1.1 Sample size 14
7.1.2 Preliminary Data Analysis Plan 15
7.1.3 Evaluation criteria: 15
8 REMOVAL OF SUBJECT FROM STUDY 15
8.1 DISCONTINUATION OF STUDY 15
8.2 WITHDRAWAL FROM STUDY 16
8.2.1 Discontinuation of Study Procedures 16
8.2.2 Early Withdrawal/Study Termination 16
9 ADVERSE EVENT REPORTING 16
9.1 DEFINITION AND GRADING INTENSITY OF ADVERSE EVENTS (AE) 16
9.2 CRITERIA FOR DETERMINING RELATIONSHIP TO STUDY PROCEDURES 16
9.3 SERIOUS ADVERSE EVENT 17
9.4 POTENTIAL RISKS AND DISCOMFORTS 17

9.4.1 Risks *17*
9.4.2 Potential benefits *18*
9.4.3 Risk:Benefit ratio *18*

10 ETHICS 18
10.1 RESPONSIBILITIES OF THE INVESTIGATOR 18
10.2 INSTITUTIONAL REVIEW BOARD APPROVAL 18

11 DATA HANDLING AND RECORDKEEPING 19
11.1 CONFIDENTIALITY 19
11.2 FLOW OF INFORMATION AND RECORDS TO BE KEPT 19

12 FINANCING 19

13 PUBLICATION POLICY 19

14 REFERENCES 20

16 APPENDICES 22
16.1 INFORMED CONSENT FORM 22
16.2 PAIN SCALE SCORE 23
16.3 HARVARD/NSABP/RTOG SCALE SCORE 24

1

Background and introduction

Breast cancer is the most common cancer of women in the world; it is estimated that 470,000 women died of breast cancer in 2008.[1] Improving breast cancer treatment outcome and survival depends on early detection and effective use of multimodality therapy: surgery, radiation oncology, and medical hormonal and chemotherapy treatments. Surgery for early stage breast carcinoma is either total mastectomy (complete breast removal) or surgical lumpectomy (tumor removal) with radiation therapy, commonly known as Breast Conserving Therapy (BCT).[2-4] The goals of BCT are to achieve local control of the cancer as well as to preserve a breast that satisfies the woman's cosmetic emotional and physical needs.[5] While most women undergo partial mastectomy with satisfactory cosmetic results, in many patients the remaining breast is left with significant cosmetic defects including concave deformities, distortion of the nipple-areola complex, asymmetry and changes in tissue consistency characterized by excessive density associated with parenchyma scarring. These flaws have been reported to contribute to poor body image and psychological distress in some patients.[5,6] Research efforts to improve the surgical outcomes of BCT in regards to prediction of cosmetic and functional outcome are very limited.

There are currently no tools, other than surgical experience and judgment that can optimize cosmetic outcome or predict the impact of partial mastectomy on the contour and deformity of the treated breast. The objectives of this study are to determine if a computational model can facilitate the prediction of the breast contour, surface features and tissue density after partial mastectomy, and potentially identify targets for intervention or suggest new procedural modifications to improve cosmetic results. An effective simulation method to predict the surgical outcome of BCT intervention would be useful for the surgeon to ensure a satisfactory cosmetic result and could be a practical tool to aid effective communication with patients.

A novel concept in surgical procedural planning will be used in this study. Wound healing after lumpectomy offers a good model to learn about the mechanism of recovery after internal tissue resection. Physical properties, such as tissue mechanics[7] and gravity, along with biological processes are factors in wound healing. While wound healing has been exhaustively studied in tissues including skin, bone, and embryos,[8-12] there is very little information about predictive models for healing after surgical resection, particularly for the breast. Currently, no satisfactory animal models exist for BCT, which prompted our initial investigations in human subjects using pre-existing images.[13]

We appear to be the first team working on a computational framework design to predict BCT outcomes.[14] This work is a good example of the converging concepts of computational surgery – http://www.computationalsurgery.org, and in particular the translation of mathematical modeling into strategies to improve clinical practice. While advances in medical imaging technologies, and informatics have contributed to advances in surgical planning, we propose there is great merit in developing computational models that can quantitatively predict surgical outcomes and offer strategies to optimize surgical procedures.

With the release of sophisticated Finite Element Modeling (FEM) software, breast modeling has been widely investigated in various applications, including biopsy planning and surgery simulation. Azar et al.[15] and Samani et al.[16] proposed a method for guiding clinical breast biopsy based on a deformable FEM of the breast. Tanner et al.[17,18] presented a method to evaluate the accuracy with which biomechanical models can predict the displacements of tissue within the breast.

Subsequently, research involving breast implants was introduced. Computer simulations for reconstructing the breast have been presented using idealized and realistic geometric models, then subsequently in actual patients. Pamplona et al. have analyzed the impact of stress and

strain occurring in the skin immediately after breast reconstruction.[19] More recent works have focused on predicting the impact of gravity loading on breast mechanics, and reversely, finding the reference state of the breast from the deformed configuration.[20,21]

In our laboratory, we have performed 3-D virtual surgery using patient MRI data and studied the cosmetic impact of BCT operation.[14] This simulation was restricted to the immediate impact of mechanical tissue removal and may predict the immediate contour result of surgery, not the final shape of the breast after healing and has not been compared to results in actual patients.

1.1 Approach to the problem

The immediate impact of tissue removal on breast deformation, as a function of tumor location and size, has been defined by our work using 3-dimensional reconstruction of the breast shape from MRI data taken with patients in a prone position. This approach uses a mechanical model that combines a hyper-elastic model of the soft tissue and a mechanical model of the skin envelope to compute breast deformation as a function of the position of the patient in two consecutive steps: position of the breast at zero gravity and at various gravity loads. From this information, a Graphical User Interface (GUI) is being developed to allow the surgeon to remove tissue in a virtual environment and visualize (or predict) the outcome. Pressures inside the wound will also impact the outcome, so an additional GUI will be developed to simulate changes that occur during treatment (eg, radiation therapy) and healing. In order to confirm the predictive validity of the multiscale model of breast lumpectomy and healing, we plan to study patients who are actively undergoing the surgical removal of breast tissue, treatment for breast cancer, and breast tissue healing.

1.2 Rationale for the clinical study

The goal of BCT is to provide a safe therapeutic alternative to total mastectomy while preserving a cosmetically satisfactory and sensate breast. The procedure is less complex and disfiguring than total mastectomy, recovery is more rapid and complications are fewer. A limitation of BCT is that in up to 25% of patients, even lumpectomy, can disfigure the breast. Patients at high risk for deformation include women with relatively small breasts, with volume loss due to previous biopsies, those with high tumor to breast size ratio, and those with tumor positioned in specific areas of the breast – such as those immediately beneath the nipple. Surgeons have recently introduced “oncoplastic” lumpectomy in an effort to move adjacent tissues into the defect created by lumpectomy at the time of initial lumpectomy.[22,23] This procedure has not been widely embraced given the unpredictable nature of the healing process, and the fact that should positive margins be identified on final pathologic evaluation, the only feasible secondary procedure is total mastectomy rather than re-excision lumpectomy.

However, accurate prediction of breast contour after lumpectomy remains an art rather than a science. The ability to reliably predict breast contour after BCT based on pre-operative imaging and “virtual” surgery would represent a significant improvement in breast cancer patient care. Patients could be fully informed regarding the outcome of surgery and radiation, allowing more evidence-based patient decision-making. Further, for those patients in whom a cosmetic tissue defect will be predicted, a secondary reconstructive procedure would be anticipated – delayed oncoplastic procedures – while still preserving a sensate and natural breast. Breast reconstruction after total mastectomy leaves patients with insensate skin, absence of the nipple-areola complex in most patients, and recovery after these multi-step procedures is much longer than after BCT. Given the equivalence of these two procedures in delivering long- term survival (BCT has been shown to be equivalent to total mastectomy for patients with non-metastatic breast cancer),[4] BCT is clearly a preferred strategy for those women who qualify for the procedure from an oncologic perspective.

The ability to accurately predict breast contour after breast conserving therapy (BCT) for breast cancer could significantly improve patient decision-making regarding the choice of surgery for breast cancer - BCT or mastectomy. As BCT is equivalent to total mastectomy to achieve overall survival in patients with non-metastatic breast cancer, the procedure of BCT offers significant advantages for the 70% of patients who are candidates for this procedure.

2 Purpose and objectives

2.1 Purpose

The purpose of this study is to test the predictive validity of the multiscale model of breast lumpectomy and healing in patients undergoing BCT for breast cancer and identify targets for improvement in BCT.

2.2 Objectives

2.2.1 Primary objective

The primary objective of this study is to compare the surgical lumpectomy, lumpectomy healing process and surface contour changes to the predicted multiscale model.

2.2.2 Secondary objective

Secondary objectives are to

- provide information regarding the use of 3-dimensional surface imaging to detect changes over time in the skin surface and dimension related to lumpectomy healing and breast contour
- provide information on the use of ultrasound of the lumpectomy bed and changes that occur during the course of breast cancer treatment and healing
- provide information on thermal imaging and inflammation symptoms
- to describe the course of pain experienced by women undergoing BCT and the correlation of pain to the healing process.

3 Design rationale

The proposed study is a single-center, prospective, pilot observation study to tabulate and measure the changes that occur during treatment and healing for BCT in women with non-metastatic breast cancer for comparison to the multiscale model of breast lumpectomy and healing in order to identify targets for improving BCT.

There is currently no information on which to base a prediction of breast contour following BCT, but the problem of cosmesis following BCT is substantial for many patients. The data collected in this observational study will provide a basis for testing the predictability of the multiscale model of breast lumpectomy and healing.

4 Experimental plan

4.1 Study design

This is a single-center, prospective, pilot observation study. Eligible subjects who enroll in this study will begin at the time of informed consent, which will occur during the pre-operative, surgical evaluation time.

The study plan demonstrates when subjects will have their surgery and when subjects will have study visits (Figure 1). The planned procedures are detailed in section 5 and the schedule of events table in section 6.

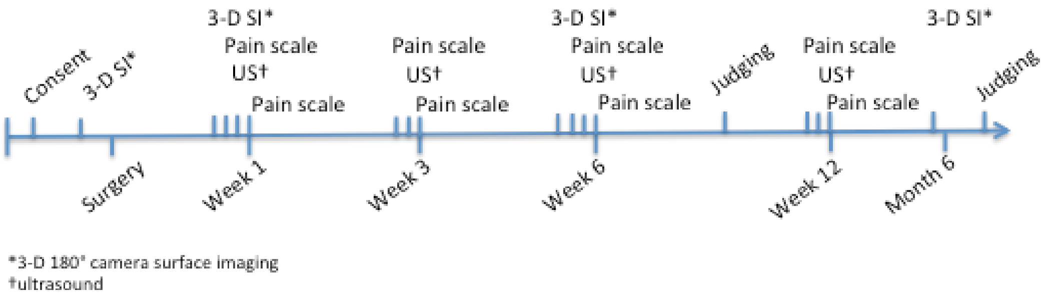

Figure 1. Study design.

4.1.1 Study endpoints

The primary endpoint of this study is the comparability of the surgical/healing outcome of the lumpectomy and breast surface contour to the predicted changes from the multiscale model. A mean combined score ≥60% agreement will be considered acceptable in this pilot project.

4.1.1.1 Primary variables

The primary variables will be surface contour captured by the 3-dimensional 180° camera, lumpectomy dimensions captured by ultrasound, predicted surface contour and predicted lumpectomy dimensions from the multiscale model.

4.1.1.2 Secondary variables

Secondary variables will be the assessment of pain over the course of the 6-month treatment and healing process, the assessment of pain associated with ultrasound (pre- ultrasound to post-ultrasound pain assessments per visit), surface temperature changes related to healing using a thermal imaging camera, and judgment of independent reviewers regarding the correlation of the cosmesis score to the multiscale prediction model.

4.1.2 Number of subjects

Up to 15 de novo breast cancer subjects will be enrolled in order to allow for early drop-outs resulting in 12 evaluable cases.

4.1.3 Study duration

The study will begin at the time of breast cancer surgery evaluation and subjects will be enrolled for six months post-surgery.

4.1.4 Study population

Patients with breast cancer who are having BCT and who satisfy the following criteria will be considered for participation in this study.

4.1.4.1 Inclusion criteria

1. Adult females ≥ age 30 years
2. Have early stage (Stage I, II), non-metastatic breast cancer
3. Planned to receive BCT
4. Have received a pre-operative mammogram within 30 days of surgery
5. Have received pre-operative magnetic resonance imaging (MRI) within 30 days of surgery
6. Planned to receive post-surgery radiotherapy by whole breast radiotherapy
7. Signed informed consent form prior to any research assessment

4.1.4.2 Exclusion criteria

1. Adult females younger than 30 years of age
2. Previous breast cancer
3. Neo-adjuvant therapy for breast cancer
4. Pregnant or nursing females
5. Participation in a study of investigational drug in the previous 30 days or 5 half-lives of the investigational drug

5 Treatment procedures

Standard of care for early stage breast cancer is breast conserving therapy at this center.

5.1 Other concomitant standard of care treatment

- Radiation therapy to the whole breast
  - 45-50 Gy delivered over 25-28 fractions with a boost to the tumor bed of 10-16 Gy delivered over 5-8 fractions

6 Study procedures

6.1 Consent process

After approval by The Methodist Hospital Research Institute's Institutional Review Board, the Principal Investigator, or a designated member of her study team, will approach potential subjects prior to surgery regarding participation in the study. The study will be described by explaining the purpose of the study, the methods and objectives and the potential risks associated with the study. An informed consent form (ICF) will be provided, patients will be given time to read the ICF and will be allowed to ask questions about the study. They will be informed that their participation is voluntary and that choosing not to be involved will not influence their care at The Methodist Hospital. They will be informed that they may choose to withdraw from the study at anytime for any reason. Subjects will be given an original informed consent form to keep. The informed consent process will be documented in the patient's chart.

Any new information about the study procedures that may be determined by the Principal Investigator to be necessary will be provided to the Institutional Review Board. Subjects will also

be provided this information through a revised informed consent form and will be given the opportunity to decide whether to continue in the study or not.

6.2 Assessment of eligibility

Within 21 days prior to surgery, the subject may be approached regarding potential interest in study involvement. Their medical history will be reviewed to assess for eligibility. Prior to performing any research-related procedures, informed consent will be obtained. Subjects must meet all inclusion criteria and none of the exclusion criteria or enrollment will not be possible.

6.3 Enrollment

Subjects who qualify for enrollment in this study, after signing informed consent, will be enrolled and proceed into the study at the time of consent. Imaging procedures and follow-up clinical appointments will be scheduled as described in Sections 6.4 through 6.7 below.

6.4 3-Dimensional Surface Imaging of the Breast

A 3-dimensional 180° camera will be used to capture surface imaging prior to surgery and at three time-points after surgery (visits 2, 4 and 6). The surface imaging will provide measures of breast deformity.

6.5 Breast Ultrasound

Breast ultrasound will be performed in the outpatient surgery clinic at visits 2, 3, 4, 5, and 6 using an office-application ultrasound system with a soft-tissue transducer, such as the SonoSite EDGE™ machine with HFL50x or HFL38x transducer.

6.6 Pain scale score

Pain will be assessed prior to surgery and at each follow-up visit prior to the ultrasound procedure and following the ultrasound procedure.

6.7 Thermal Imaging of the Breast

The healing process will be assessed by taking surface images with a thermal camera.

6.8 Judging

Breast cosmesis will also be assessed using the 4-point Harvard/NSABP/RTOG cosmesis criteria scale (Appendix) and judged by two independent surgeons not involved in the care of the patient or the study. Judging will occur at baseline (after surgery) and at week 10 and week 26.

6.8.1 Sequence of events

Month			**1**				**3**	**6**	
Week			1	3	6	10	12	24	26
Visit day	-21 to 0		1	2	3		4	5	
Visit window (days)	-21		0	±7	±7		±7	±7	
Visit number	1		2	3	4		5	6	
Parameter									
Informed consent	X								
3-D SI*	X		X		X			X	
Surgery		X							
Thermal image			X	X	X		X	X	
Ultrasound†			X	X	X		X	X	
Radiation therapy			X	X	X				
Pain scale score‡	X		XX	XX	XX		XX	XX	
Cosmesis judging§			X			X			X

*3-dimensional 180° camera surface imaging
†Breast ultrasound of the tumor / lumpectomy area
‡Performed pre and post ultrasound
§Independent assessment of cosmesis using the Harvard/NSABP/RTOG cosmesis criteria and comparison to multiscale model

6.8.2 Screening and eligibility

Between day -21 and day of surgery, after obtaining informed consent (as described in Section 6.1), subjects who fulfill all of the inclusion criteria and none of the exclusion criteria will be accepted into the study. Documentation of the fulfillment of criteria will be included in the study files.

6.8.3 Study completion

Subjects who attend the 6-month study visit and have completed all study-related evaluations described in Section 6.5.2 will be considered as having completed the study.

Subjects who elect to discontinue pre-maturely or are withdrawn from the study by the investigator, will be converted to standard of care treatment alone (no further study imaging/ultrasound). If it is not possible to complete the end-of-study visit, every attempt will be made to determine safety endpoints (adverse events, breast cancer recovery status) occurring since the previous visit. A phone visit may be utilized for this information if the subject is unable or unwilling to attend the next or last study visit.

Subjects will be followed as standard of care after the study regimen has been completed or discontinued.

7 Statistical analysis plan

7.1.1 Sample size

This single-center study will enroll up to 15 de novo breast conserving therapy subjects. This is a prospective, pilot, observational study. Subjects withdrawn from the study cannot re-enter. Up to 3 patients, if withdrawn prior to week 6, will be replaced. Twelve subjects will be considered

evaluable if they complete up to visit 5 and will be considered completers if they complete all study visits and procedures.

7.1.2 Preliminary Data Analysis Plan

Evaluable subjects will have completed a minimum of 5 visits (week 6). Descriptive statistics will be used to summarize the demographic and clinical characteristics of subjects. Changes at the end of study from baseline will be evaluated using chi-square for categorical variables and Kruskal-Wallis for continuous variables.

Receiver operator characteristic curves will be used to explore the sensitivity of the multiscale model to the outcome.

7.1.3 Evaluation criteria:

- The primary outcome measure will be
- Qualitative:
  - confirmation that relative size of the lumpectomy to the breast, tissue composition ratio of fat versus glandular, and location of the tumor will provide a qualitative grading of the potential cosmetic outcome.
  - correlation of the wound edge shape to mechanical stress
  - correlation of volume loss due to healing to cosmesis outcome.
- Quantitative: a combined score of the correlation between three measured processes (lumpectomy dimensions, lumpectomy healing process, surface contour changes) and the predicted outcomes from the multiscale model measured/assessed at weeks 10 and 26 postsurgery with a mean accuracy rate of ≥60%.
- Secondary outcome measures will include postsurgery:
  - the proportion of subjects experiencing pain ≥8 on more than one measurement post operatively and the relationship of pain to the accuracy of the multiscale model
  - the proportion of subjects experiencing increased pain (≥3 unit increase) pre to post ultrasound at each visit to determine the possible invasiveness of these multiple post-surgery ultrasounds
  - detection of changes in the thermal image of the breast tissue related to inflammation and healing
  - the summary of the independent judgment (Harvard/NSABP/RTOG scale score) of cosmesis taken at weeks 10 and 26

8 Removal of Subject from Study

8.1 Discontinuation of Study

The Principal Investigator reserves the right to discontinue this study at any time. Should this occur, the appropriate authorities (eg, Institutional Review Board) will be informed, and the reason for discontinuation will be indicated in the Case Report Form (CRF). A study termination CRF must be completed.

8.2 Withdrawal from Study

8.2.1 Discontinuation of Study Procedures

The Principal Investigator will discontinue testing a subject if she believes it is in the subject's best interest. When a subject discontinues receiving imaging studies, the subject will be followed according to the standard of care. The date that the subject discontinues the study will be recorded.

8.2.2 Early Withdrawal/Study Termination

Subjects who enroll in the protocol and withdraw, at any time for any reason, before visit 5 are considered early withdrawals and will be replaced up to a maximum of 3 withdrawals. A complete set of data including reason for withdrawal will be collected on each subject through the time interval of withdrawal.

Subject tracking should include, but is not limited to: (1) at least 3 attempts to contact subjects by phone, (2) at least 3 attempts to contact subjects through the names subjects authorized, and (3) 1 attempt to contact subjects by registered letter. Documentation of all contact attempts should be retained in the source documents for the subject, including a copy of the letter sent and the returned receipt of mailing.

Subjects experiencing adverse reactions should be followed until the reaction has resolved, stabilized, or is otherwise explained.

9 Adverse event reporting

9.1 Definition and Grading Intensity of Adverse Events (AE)

An AE is defined as any unintended change in the body structure (signs) or body function (symptoms), whether or not considered study-related. During confinement, subjects will be instructed to report all adverse events. All AEs, whether volunteered, elicited, or noted on physical examination, will be recorded throughout the study, eg, from the time of dose administration until study completion/discharge.

In addition, rating based on the National Cancer Institute (NCI) Common Toxicity Criteria (version 4.0, http://ctep.cancer.gov/forms/CTCAEv4.pdf), the AE intensity and changes over time will also be characterized as mild, moderate, severe, not related, possibly related, probably related, or definitely related based on the following definitions:

- Mild: Experience is minor and does not cause significant discomfort to subject or change to activities of daily living; subject is aware of symptoms but symptoms are easily tolerated.
- Moderate: Experience is an inconvenience or concern to the subject and causes interference with the activities of daily living.
- Severe: Experience significant interference with activities of daily living and the subject is incapacitated and/or unable to continue with activities of daily living.

9.2 Criteria for Determining Relationship to study procedures

The clinical investigator will make a determination of the relationship of the AE to the study procedures using a four-category system (not related, possibly related, probably related, definite).

Not related: Any untoward AE that either was clearly pre-existent, or occurred at the time of test procedures but sufficient information exists to indicate the etiology is unrelated to the clinical procedure.

Possibly related: An AE that does not follow a reasonable temporal sequence from the test procedure, does not follow a known or suspected response pattern, or could be explained by another etiology.

Probably related: An AE that follows a reasonable temporal sequence from the test procedure, follows a known or suspected pattern of response to the procedure, and cannot be reasonably explained by the known characteristics of the subject's clinical state.

Definitely related: An AE that follows a reasonable temporal sequence from test procedure and there is no other cause to explain the event, or a re-challenge (if feasible) is positive

9.3 Serious adverse event

A serious Adverse Event (SAE) is any adverse event occurring at any dose that results in any of the following outcomes:

- Death;
- Life-threatening adverse experience;
- Inpatient hospitalization or prolongation of existing hospitalization;
- Persistent or significant disability/incapacity;
- Congenital anomaly/birth defect; and.
- Important medical events that may not result in death, be life-threatening, or require hospitalization may be considered a serious adverse event when, based upon the appropriate medical judgment, they may jeopardize the subject and may require medical or surgical intervention to prevent one of the outcomes listed in this definition.

Adverse events that fulfill criteria for Serious Adverse Event (SAE) will be reported to the sponsor within 24 hours of the known event and to the Institutional Review Board within 72 hours of the known event.

Hospitalizations for radiation therapy, should they occur, will not be considered as SAEs. Likewise, planned or elective procedures for pre-existing conditions (known prior to first administration of study drug) that are not related to a worsening of the condition after study drug initiation will not be considered as SAEs.

9.4 Potential risks and discomforts

9.4.1 Risks

9.4.1.1 3-Dimensional Camera Imaging

There are no risks associated with the 3-dimensional 180° camera surface imaging. The camera will not be in contact with the subject’s skin.

9.4.1.2 Ultrasound

There are no known long-term side effects of ultrasound. The ultrasound procedure is not expected to cause discomfort to the patient but pain will be assessed/quantified pre/post ultrasound at each visit in order to capture this information. If at any time the subject expresses the desire to discontinue the ultrasound procedure, they will be allowed to discontinue the procedure.

9.4.1.3 Thermal imaging

There are no risks associated with the thermal imaging camera. The camera will not be in contact with the subject's skin.

9.4.1.4 Risks associated with radiation therapy

Radiation therapy risks are noted as part of the protocol, but radiation procedures are not an experimental component of this protocol; they are standard of care for BCT. Long-term effects include radiation pneumonitis, rib fractures, and (if the left breast is the side to receive radiation) cardiac complications. Short-term side effects of radiation therapy include fatigue, skin erythema, desquamation, breast edema, breast tenderness and myositis. Reporting of radiation therapy side effects may be important in this study because we do not know how they will impact the accuracy of the multiscale model.

9.4.2 Potential benefits

This study will improve the knowledge of clinicians who perform BCT. It will provide needed information for improving the BCT procedure if the multiscale model is successful. But subjects participating in this pilot study will not benefit directly from this research.

9.4.3 Risk:Benefit ratio

The benefit:risk for participants in this study is considered mildly negative given that the subject is not expected to benefit directly from the research and might experience mild pain during the ultrasound procedures. Participating in the study may be perceived as an inconvenience to some patients.

10 Ethics

10.1 Responsibilities of the investigator

The Principal Investigator will maintain complete study files (electronic and/or paper) to enable full documentation of the study procedures and data for purpose of verification and study analysis.

The Principal Investigator, or her designee, will explain the nature of the study to the subject, inform the subject that participation is voluntary and that withdrawal at any time is allowed. The Principal Investigator will maintain documentation of written informed consent.

This study will be conducted using Good Clinical Practice as identified by

- International Conference on Harmonization (ICH) Tripartite Guidelines for Good Clinical Practice
- US Code of Federal Regulation (CFR) dealing with clinical studies (21 CFR parts 50, 54, 56 and 312)
- The principles stated in the Declaration of Helsinki concerning medical research in humans

10.2 Institutional review board approval

This study will be reviewed and approved by The Methodist Hospital Research Institute Institutional Review Board (TMHRI IRB) prior to commencement of any subject-specific activities. A current letter of IRB approval will be maintained with the study files. No changes to

the protocol or informed consent form will be made prior to or without documented approval from TMHRI IRB.

All subjects will provide consent (indicated by their signature or their legally authorized representative's signature on the approved Informed Consent Form) prior to initiation of any study-related activities. Signed and dated ICFs will be maintained with the study archives.

11 Data handling and recordkeeping

11.1 Confidentiality

In order to maintain confidentiality, the subject will be identified only by his/her study screening number and/or randomization number.

11.2 Flow of information and records to be kept

Throughout the conduct of this study, all required data will be recorded in electronic CRFs that have been specifically designed to record all observations and other data pertinent to this clinical investigation. Data reported on CRFs should be consistent with source documents when applicable, or the discrepancies should be explained. Any change or correction to a CRF will produce an audit trail within the electronic CRF system and will identify the user, date and time of the change.

The CRFs should be completed in a timely fashion. The complete electronic CRFs will be maintained by the study site, as is required by institutional, local and government regulations.

The clinical investigator is responsible for maintaining adequate and accurate records as specified in Essential Documents for the Conduct of a Clinical Trail (section 8 of the ICH Guideline for Good Clinical Practice) to enable the conduct of the study to be fully documented and the study data to be subsequently verified.

Upon study termination, original documents will be maintained at TMHRI.

12 Financing

Funding for this study is provided by The Methodist Hospital Department of Surgery.

13 Publication policy

The Principal Investigator holds final approving authority over whether or not any proposed publication (eg, abstract, manuscript) based on the data generated from this study can or should be submitted for publication to journals, scientific meetings, or any other medium.

14 References

1. Cancer. *World Health Organization Media Centre Fact Sheets* 2011; Breast Cancer Deaths. Available at: http://www.who.int/mediacentre/factsheets/fs297/en/index.html. Accessed December 14, 2011.
2. Benson J, Jatoi I, Keisch M, Esteva F, Makris A, Jordan V. Early breast cancer. *Lancet.* 2009;373(9673):1463-1479.
3. Clarke M, Collins R, Darby S, et al. Effects of radiotherapy and of differences in the extent of surgery for early breast cancer on local recurrence and 15-year survival: an overview of the randomised trials. *Lancet.* 2005;366(9503):2087-2106.
4. Fisher B, Anderson S, Bryant J, et al. Twenty-year follow-up of a randomized trial comparing total mastectomy, lumpectomy, and lumpectomy plus irradiation for the treatment of invasive breast cancer. *N Engl J Med.* 2002;347(16):1233-1241.
5. Waljee JF, Hu ES, Ubel PA, Smith DM, Newman LA, Alderman AK. Effect of esthetic outcome after breast-conserving surgery on psychosocial functioning and quality of life. *J Clin Oncol.* 2008;26:3331-3337.
6. Al-Ghazal S, Fallowfield L, Blamey R. Does cosmetic outcome from treatment of primary breast cancer influence psychosocial morbidity? *Eur J Surg Oncol.* 1999;25(6):571-573.
7. Rodriguez E, Hoger A, McCulloch A. Stress-dependent finite growth in soft elastic tissues. *J Biomech.* 1994;27(4):455-467.
8. Javierre E, Vermolen F, Vuik C, van der Zwaag S. A mathematical analysis of physiological and morphological aspects of wound closure. *J Math Biol.* 2009;59(5):605-630.
9. Grinnell F. Fibroblasts, myofibroblasts, and wound contraction. *J Cell Biol.* 1994;124(4):401-404.
10. Sherratt J, Murray J. Models of epidermal wound healing. *Proc Biol Sci.* 1990;241(1300):29-36.
11. Dale P, Sherratt J, Maini P. A mathematical model for collagen fibre formation during foetal and adult dermal wound healing. *Proc Biol Sci.* 1996;263(1370):653-660.
12. Geris L, Gerisch A, Schugart R. Mathematical modeling in wound healing, bone regeneration and tissue engineering. *Acta Biotheor.* 2010;58(4):355-367.
13. Kim J, O'Hare M, Stein R. Models of breast cancer: is merging human and animal models the future? *Breast Cancer Res.* 2004;6(1):22-30.
14. Thanoon D, Garbey M, Kim N, Bass B. A computational framework for breast surgery: application to breast conservative therapy. In: Garbey M, Bass B, Collet C, Mathelin Md, Tran-Son-Tay R, eds. *Computational Surgery and Dual Training*. 1st ed: Springer; 2010:249-268.
15. Azar F, Maetaxas D, Schnall M. A finite model of the breast for predicting mechanical deformations during interventional procedures. *Proc Int Soc Magn Reson Med Sci Meet Exhib Int Soc Magn Reson Med Sci Meet Exhib.* 1999;7:1084.
16. Samani A, Bishop J, Yaffe M, Plewes D. Biomechanical 3-D finite element modeling of the human breast using MRI data. *IEEE Trans Med Imaging.* 2001;20(4):271-279.
17. Tanner C, Schnabel J, Hill D, Hawkes D, Leach M, Hose D. Factors influencing the accuracy of biomechanical breast models. *Med Phys.* 2006;33(6):1758-1769.
18. Schnabel J, Tanner C, Castellano-Smith A, et al. Validation of nonrigid image registration using finite-element methods: application to breast MR images. *IEEE Trans Med Imaging.* 2003;22(2):238-247.
19. Pamplona D, de Abreu Alvim C. Breast reconstruction with expanders and implants: a numerical analysis. *Artif Organs.* 2004;28(4):353-356.

20. Chung J, Rajagopal V, Nielsen P, Nash M. Modelling mammographic compression of the breast. *Med Image Comput Comput Assist Interv.* 2008;11:758-765.

21. Rajagopal V, Lee A, Chung J, et al. Towards tracking breast cancer across medical images using subject-specific biomechanical models. *Med Image Comput Comput Assist Interv.* 2007;10:651-658.

22. Roughton M, Shenaq D, Jaskowiak N, Park J, Song D. Optimizing delivery of breast conserving therapy: a multidisciplinary approach to oncoplastic surgery. *Ann Plast Surg.* 2011:[Epub ahead of print] Aug 22.

23. Veiga DF, Veiga-Filho J, Ribeiro LM, et al. Quality-of-life and self-esteem outcomes after oncoplastic breast-conserving surgery. *Plast Reconstr Surg.* 2010;125:811-817.

24. Trombetta M, Julian T, Werts E, et al. Comparison of conservative management techniques in the re-treatment of ipsilateral breast tumor recurrence. *Brachytherapy.* 2011;10:74-80.

16 Appendices

16.1 Informed Consent Form

16.2 Pain Scale Score

Subject Initials: ____________________ Date: ____________________ Visit:___________ Pre or Post (circle) Ultrasound

0-10 Numeric Pain Intensity Scale*

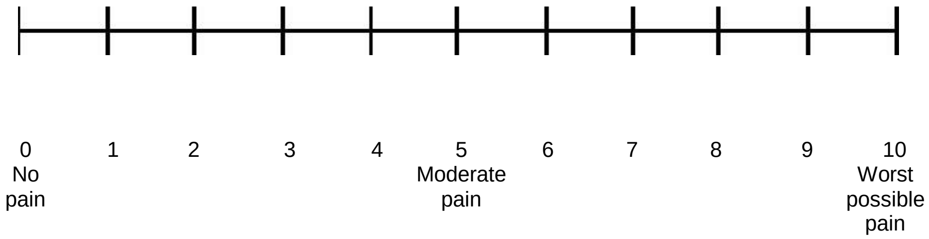

*If used as a graphic rating scale, a 10-cm baseline is recommended.
From: Acute Pain Management: Operative or Medical Procedures and Trauma, Clinical Practice Guideline No. 1. AHCPR Publication No. 92-0032; February 1992. Agency for Healthcare Research & Quality, Rockville, MD; pages 116-117.

A7012-AS-2

16.3 Harvard/NSABP/RTOG scale score

The Harvard/NSABP/RTOG scale score is a 4-point scale of breast cosmesis, which will be assessed by clinicians at baseline (after surgery) and two additional times during follow-up (week 10 and week 26).

Harvard/National Surgical Breast and Bowel Project/Radiation Therapy Oncology Group Breast Cosmesis Grading Scale[24]

"I. Excellent: When compared with the untreated breast, there is minimal or no difference in the size or shape of the treated breast. The way the breasts feel (its texture) is the same or slightly different. There may be thickening, scar tissue, or fluid accumulation within the breast but not enough to change the appearance.

"II. Good: There is a slight difference in the size or shape of the treated breast as compared with the opposite breast or the original appearance of the treated breast. There may be some mild reddening or darkening of the breast. The thickening or scar tissue within the breast causes only a mild change in the shape or size.

"III. Fair: Obvious difference in the size and shape of the treated breast. This change involves one-quarter or less of the breast. There can be moderate thickening or scar tissue of the skin and the breast, and there may be obvious color changes.

"IV. Poor: Marked change in the appearance of the treated breast involving more than one-quarter of the breast tissue. The skin changes may be obvious and detract from the appearance of the breast. Severe scarring and thickening of the breast, which clearly alter the appearance of the breast, may be found."
